# A Species‐Dependent Spatial Patterning of FOXP1 and nNOS Protein Expression in the Adult Mammalian Subthalamic Nucleus

**DOI:** 10.1002/cne.70219

**Published:** 2026-10-28

**Authors:** Tila Medenica, Ema Bokulić, Miloš Judaš, Goran Sedmak

**Affiliations:** ^1^ University of Zagreb School of Medicine, Croatian Institute for Brain Research Zagreb Croatia; ^2^ Centre of Excellence for Basic, Translational and Clinical Neuroscience Zagreb Croatia

**Keywords:** FOXP1, HuC/HuD, human, mouse, nNOS, rat, subthalamic nucleus

## Abstract

The subthalamic nucleus (STN) is a key component of basal ganglia circuitry involved in motor, cognitive, and limbic functions. While the transcription factor FOXP1 has been implicated in neurodevelopmental disorders and basal ganglia function, its expression in the adult STN remains insufficiently characterized. Our study investigated the expression and distribution of FOXP1 protein and its colocalization with neuronal nitric oxide synthase (nNOS) and a pan‐neuronal marker (HuC/HuD) in the adult STN across three mammalian species: mouse, rat, and human. Using immunofluorescent double‐labeling and quantitative analysis, we examined their spatial organization and relative proportions within the neuronal population. FOXP1 was identified as a substantial neuronal subpopulation, comprising approximately 40%–45% of STN neurons across all species. In contrast, nNOS‐positive neurons showed marked interspecies variation, increasing from ∼32% in mice to ∼68% in humans. Notably, the spatial distribution of these markers differed significantly between species. In rodents, FOXP1+ and nNOS+ neurons were largely segregated along the dorsoventral axis, forming visibly distinct subdomains. Conversely, in the human STN, these populations were diffusely intermixed with a higher degree of colocalization. Additionally, both markers exhibited species‐specific antero‐posterior gradients. These findings provide the first evidence of FOXP1 protein expression in the adult rat and human STN and highlight fundamental interspecies differences in neuronal organization. The expanded nNOS+ population and increased FOXP1/nNOS colocalization in humans suggest evolutionary divergence in the mammalian STN cytoarchitecture. Overall, the results challenge simplified models of STN subdivision in mammals, primates especially, and underscore the need for caution when extrapolating rodent data to human neurophysiology.

## Graphical abstract

FOXP1+ and nNOS+ neurons constitute distinct neuronal populations in the adult subthalamic nucleus, with spatial segregation in rodents but extensive intermixing in humans. The human STN also shows an expanded nNOS+ population and increased FOXP1+/nNOS+ colocalization, highlighting species‐specific differences in STN organization.

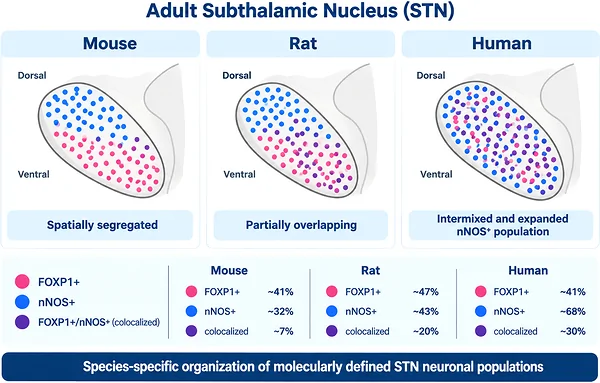

## 1 Introduction

The subthalamic nucleus (STN) is a small subcortical structure that is an important part of the basal ganglia circuitry (Parent and Hazrati 1995; Grillner and Robertson 2016). Besides its established role in motor control, STN is significantly involved in cognitive and limbic processes in the brain (Temel et al. 2005; Baunez and Lardeux 2011).

FOXP1 is a transcription factor that belongs to the Forkhead box gene family, which consists of four proteins (FOXP1, FOXP2, FOXP3, FOXP4). Three of these are expressed in the mammalian brain (FOXP1, FOXP2, and FOXP4; Co et al. 2020). Animal research on the whole‐body FOXP1 loss‐of‐function showed that homozygous Foxp1 (Foxp1−/−) knockout mice die at E14.5 due to severe defects in cardiac morphogenesis (Wang et al. 2004). Heterozygous Foxp1 knockout (Foxp1+/−) neonatal mice exhibit abnormal ultrasonic vocalizations, while as adults they exhibit impaired grip strength and hyperactivity (Araujo et al. 2015), accompanied by a reduction in FOXP1 expression and a dysregulation of autism‐associated genes in the hippocampus and striatum (Araujo et al. 2015). Bacon et al. (2015) used a brain‐specific conditional homozygous knockout of Foxp1 mice (Foxp1−/−), which were viable as the brain‐specific deletion avoided heart defects. These mice, as neonates, showed abnormal ultrasonic vocalizations followed in adulthood by abnormal behavior—hyperactivity, increased repetitive behaviors, impaired short‐term memory, impaired social behavior, reduced anxiety, and impaired attentional processing, along with distinct morphological abnormalities in the hippocampus and striatum (Bacon et al. 2015).

In humans, disrupting mutations in the Foxp1 gene, namely, deletions and duplications, can cause the FOXP1 syndrome—a neurodevelopmental disorder with a wide array of cognitive, motor, and behavioral symptoms such as developmental and speech delay, motor deficits, sleep problems, and intellectual disability (Meerschaut et al. 2017; Siper et al. 2017; Co et al. 2020; Lozano et al. 2021). Affected individuals can have several psychiatric symptoms: attention deficit/hyperactivity disorder (ADHD) symptoms such as inattention, impulsivity, and hyperactivity; aggression, obsessive–compulsive symptoms, mood disorders, anxiety; and autism spectrum disorder symptoms such as social skills deficits, sensory symptoms, and repetitive behaviors or interests (Lozano et al. 2021).

The majority of FOXP1 disruption behavioral symptoms can be linked to (dys)functions of the STN, such as inattention (Adey et al. 1962; Baunez and Robbins 1997; Baunez et al. 2007; Lindsley et al. 1970; Chudasama et al. 2003), impulsivity (Baunez and Robbins 1997; Baunez et al. 2007; Brittain et al. 2012; Frank et al. 2007), aggression (Choi et al. 2018; Papuć et al. 2014 Sensi et al. 2004), and repetitive behaviors (Mallet et al. 2008; Polosan et al. 2019; Voon et al. 2017; Parolari et al. 2021); however, the data on FOXP1 expression and function in the STN are scarce. In the developing and adult mouse brain, Foxp1 mRNA expression was found by Ferland et al. (2003) in basal ganglia structures such as the striatum and substantia nigra, but there were no reports on its expression in the STN. Tamura et al. (2004) reported the absence of Foxp1 mRNA signal in the adult mouse STN; however, in an RNA‐sequencing study, Foxp1 mRNA in the mouse STN was detected (Wallen‐Mackenzie et al. 2020), while Skidmore et al. (2008) reported FOXP1 protein expression in the mouse STN detected by immunofluorescence. Foxp1 mRNA was found in the STN of 22 gestational weeks old human fetal brains (Teramitsu et al. 2004), and so was FOXP1 protein in the human fetal STN as early as 13 gestational weeks (Bokulić et al. 2025). It seems that the expression of FOXP1 in the developing human STN is supported by available data on the transcriptional and translational level; however there are no reports on the FOXP1 expression in the adult human STN, and reports on the expression in adult mouse STN are not entirely consistent. Furthermore, there is no data for FOXP1 expression in the rat STN, an important model species in neuroscience (Ellenbroek and Youn 2016). Due to this, the first aim of this paper is to investigate and compare the expression and distribution of the FOXP1 protein across species in adult human, mouse, and rat STN.

A protein that is described more thoroughly across species regarding its expression in the STN is the neuronal nitric oxide synthase (nNOS, encoded by Nos1). About 95% of human subthalamic neurons express mRNA for Nos1 (Nisbet et al. 1994), while about 90% express the protein nNOS itself (Santos‐Lobato et al. 2016; Bokulić et al. 2021). Vincent and Kimura (1992) used the NADPH‐diaphorase histochemical technique to map nNOS in the rat brain and reported that “The cells of the subthalamic nucleus were very weakly stained,” while De Vente et al. (1998) used immunostaining to map nNOS in the rat brain and observed “numerous NOS‐positive cell bodies” in the STN. Immunostaining of the subthalamic nNOS‐positive cells was also reported in the adult mouse brain (Cavalcanti‐Kwiatkoski et al. 2010). The second aim of the paper is to determine the colocalization of the FOXP1 protein with nNOS, as well as with a pan‐neuronal marker HuC/HuD (Akamatsu et al. 2005; Pascale et al. 2008), and to compare their expression profiles and distribution in the three mammalian species (mouse, rat, and human).

## 2 Materials and Methods

### 2.1 Tissue Acquisition and Preparation

In this study, we used STN samples from adult specimens of three species: mouse, rat, and human. Rodent samples were collected by educated and certified staff from the animal breeding facility of the Croatian Institute for Brain Research, in accordance with guidelines on animal welfare and with prior approval from the Ethical Committee of the University of Zagreb School of Medicine and the Institutional Review Board. Animals were housed in polysulfone cages in a controlled temperature (21 ± 2°C) and humidity (65 ± 5%) environment. A light/dark cycle of 12:12 h was employed with the light period beginning at 7 a.m. Food (4RF21C, Mucedola srl, Settimo Milanese MI, Italy) and tap water were provided ad libitum.

The STN was sampled from three 3.5‐month‐old adult male Wistar Han rats (RccHan: WIST) and three 3.5‐month‐old male C57BL/6 mice. Rodent brains were obtained by performing transcardial perfusion fixation procedures according to the protocol established by Gage et al. (2012). Prior to the procedure, the animals were deeply anesthetized with an intraperitoneal administration mixture of 80–100 mg/kg ketamine and 10 mg/kg xylazine (both from Bioveta, Czech Republic) in isotonic solution. Perfusion was done with ice‐cold phosphate‐buffered saline (PBS) first, and subsequently with 4% formaldehyde. After extraction, all brains were immersion‐fixed in 4% paraformaldehyde in 0.1 M phosphate‐buffer solution (PBS, pH = 7.4).

The three adult human brain specimens belong to the Zagreb Collection of human brains (Judaš et al. 2011). The specimens were collected during routine autopsies at the Department of Forensic Medicine of the University of Zagreb School of Medicine, with the prior approval of the Ethical Committee of the University of Zagreb School of Medicine. The sampling is approved by next of kin through signing the informed consent. The informed consent form is approved by the Ethical Committee of the University of Zagreb School of Medicine. Upon entering the Collection, brain samples get labeled with numbered ID codes and donor names are anonymized. The brains used in this study did not exhibit any macroscopic or microscopic pathology (Table 1). The brains were immersion‐fixed in 4% paraformaldehyde in 0.1 M phosphate‐buffer solution (PBS, pH = 7.4) for 2 weeks. After fixation, the brains were divided into hemispheres, and each hemisphere was cut coronally into 2–3 cm thick slabs. The tissue slab that contained the STN was further dissected to isolate the STN into one small tissue block used for subsequent experiments. These smaller tissue blocks were postfixed until complete fixation for up to 2 additional weeks.

**TABLE 1:** Metadata on human brains used in the study.

Subject no.	Gender	Age range	Cause of death	Race	Postmortem delay (h)
H1 (383)	M	51–55	Heart attack	Caucasian	11
H2 (394)	M	61–65	Asphyxiation	Caucasian	12
H3 (395)	M	56–60	Pneumonia	Caucasian	24

All specimens were without microscopic or macroscopic pathological changes. As seen by the cause of death no specimen underwent a prolonged period of agonal state.

Following fixation, all STN samples were dehydrated through a graded series of alcohol solutions (70%, 96%, and 100%), cleared through toluene immersion, and finally paraffin‐embedded. Embedded tissue blocks were cut on a microtome (Leica SM 2000R, Leica Biosystems) into 10 µm‐thick sections. Nissl staining was used to determine the span of sections containing the STN. Prior to the staining, the slides were deparaffinized in xylene. The human sample slides went through an additional step of submersion in the chloroform–ether solution (eight volumes of chloroform, one volume of ether, and one volume of 96% ethanol) for 5 min, left to dry, and washed in dH_2_O to enhance the tissue adhesion to the slides. Following deparaffinization and the subsequent rehydration in the graded series of alcohol solutions, all slides were immersed in the 0.5% Cresyl–Violet solution and visually inspected for the quality of staining. Following differentiation and decolorization in the solution of 70% ethanol with or three drops of acetic acid, the slides were coverslipped with Histomount (National Diagnostics, USA).

### 2.2 Antibody Characterization

The FOXP1 antibody (Abcam, ab16645, RRID: AB_732428) is a rabbit polyclonal antibody made against a synthetic peptide that corresponds to the C‐terminus of human FOXP1 conjugated to Keyhole Limpet Hemocyanin (KLH). The antibody detects a band of approximately 75 kDa on Western blots of mouse embryo lysates, as well as additional bands of different isoforms of FOXP1 protein (manufacturer's datasheet). The antibody was previously used on a number of occasions to detect mouse FOXP1 protein (Wang et al. 2011; Merchan‐Sala et al. 2017; Tinterri et al. 2018; Tulloch et al. 2019), showing consistent staining and distribution patterns. Additionally, the antibody was validated by Bowers et al. (2013), who showed that the FOXP1 antibody detects 75 kDa bands on Western blots of rat and human brain tissue lysates, and by Sheng et al. (2019) in human cell cultures who demonstrated that the antibody cannot detect the band in cell samples with the FOXP1 gene silenced, while it detects it normally in untreated cells (Sheng et al. 2019).

The Nitric Oxide Synthase I antiserum (Chemicon, AB5380, RRID: AB_91824) is a rabbit polyclonal antibody raised against recombinant human nNOS. The antibody recognizes nNOS and is validated for use in immunohistochemistry and Western blot—it shows no cross reaction with iNOS and eNOS by immunohistochemistry (manufacturer's datasheet). The antibody detects a band of 140 kDa on Western blots of rat neural cell lysates (Mannella et al. 2009) and a band of 170 kDa on Western blots of mouse brain lysates (Shi et al. 2022). The antibody detects three nNOS splice variants, including nNOS‐μ, nNOS‐α, and nNOS‐2, at 165, 160, and 144 kDa, respectively, on Western blots of human neuroblastoma lysates (Xia and Krukoff 2004).

The NOS1 (G‐4) antibody (Santa Cruz Biotechnology, sc‐398843) is a mouse monoclonal antibody specific for an epitope mapping between amino acids 37–56 near the N‐terminus of NOS1 of human origin (manufacturer's datasheet). The antibody is suitable for the detection of NOS1 of mouse, rat, and human origin by Western blotting and immunohistochemistry/immunofluorescence, and on Western blots of mouse and human tissue, it detects bands of around 155 kDa (manufacturer's datasheet). The antibody was used in Western blot experiments to label nNOS protein in rat tissue extracts, detecting a band of 155 kDa (Ruze et al. 2023).

The HuC/HuD (16A11) antibody (Invitrogen, A‐21271, RRID: AB_221448) is a mouse monoclonal antibody raised against the Hu family of human neuronal RNA binding proteins (HuD, HuC, and Hel N‐1), with the immunogen being the C‐terminus of the HuD protein (QAQRFRLDNLLN‐C)–(KLH) conjugate (Marusich et al. 1994). Anti‐Hu antibodies bind specifically to antigens present exclusively in neuronal cells and are thus useful as markers of neuronal cells in tissue (manufacturer's datasheet). According to the manufacturer, the antibody recognizes the Hu family members HuC, HuD, and Hel‐N1 neuronal proteins. It does not recognize HuR, another Hu family member that is present in all proliferating cells. In immunoblots performed on human, mouse, avian, and zebrafish tissue extracts, the antibody recognizes specific bands of ∼40 kDa corresponding to the molecular weights of the Hu proteins (Marusich et al. 1994), indicating that it may be reactive across all vertebrates. The specificity of the 16A11 antibody binding in immunoblots was confirmed by blocking it with the HuD peptide (Marusich et al. 1994).

### 2.3 Immunofluorescence

We performed immunofluorescent double‐labeling with antibodies for FOXP1 and nNOS proteins described in the previous chapter. Colabeling with HuC/HuD antibody (a pan‐neuronal marker) was performed to count and estimate the percentage of immunoreactive (IR) neurons in the whole neuronal population.

The sections were deparaffinized in xylol, rehydrated in a graded series of alcohol solutions, immersed in distilled water, and then the heat‐induced antigen retrieval was performed in 20 mM Tris buffer (pH 9.0) with 0.05% Tween 20 (Sigma). Afterward, sections were washed in PBS and then pretreated with methanol and hydrogen peroxide solution for 20 min. After washing in PBS, sections were blocked by a solution of 5% normal donkey serum (Sigma), 1% bovine serum albumin (Sigma), and 0.5% Triton X‐100 (Sigma) in PBS for 1 h at room temperature. Primary antibodies were diluted in the same blocking solution (Table 2), and sections were incubated with primary antibodies overnight at 4°C.

**TABLE 2:** List of primary and secondary antibodies, and their dilutions, used in the study.

Antibody	Host, clonality	Supplier	Catalog number	Dilution
*Primary antibodies*				
Anti‐HuC/HuD	Mouse monoclonal	Thermo Fisher Scientific	A‐21271	1:500
Anti‐FOXP1	Rabbit polyclonal	Abcam	ab16645	1:300
Anti‐NOS1	Mouse monoclonal	Santa Cruz Biotechnology	sc‐398843	1:50
Anti‐NOS1	Rabbit polyclonal	Chemicon	AB5380	1:800
*Secondary antibodies*				
AlexaFluor 555	Donkey anti‐mouse	Thermo Fisher Scientific	A‐31570	1:1000
AlexaFluor 488	Donkey anti‐rabbit	Thermo Fisher Scientific	A‐21206	1:1000

The next day, sections were washed 3 × 5 min in PBS and incubated with a secondary antibody solution for 2 h at room temperature. Negative controls for the secondary antibodies were used by omitting the primary antibody. We used AlexaFluor 488 donkey antibody as an anti‐rabbit antibody (Invitrogen, A21206), and as an anti‐mouse antibody, we used AlexaFluor 555 donkey antibody (Invitrogen, A31570). Secondary antibodies were diluted 1:1000 in the blocking solution containing 2.5% normal donkey serum, 0.5% bovine serum albumin, and 0.25% Triton X‐100 in PBS.

After incubation, sections were washed 3 × 5 min in PBS and then treated with TrueBlack Lipofuscin Autofluorescence Quencher (Biotium, 23007). Sections were then washed again and coverslipped with a Vectashield Antifade Mounting Medium with DAPI (Vector Laboratories, Inc.).

### 2.4 Visualization and Quantification

Fluorescent images were obtained using a laser scanning confocal microscope Olympus FV3000, using FV31S‐SW Fluoview software. Acquired fluorescent images were digitally traced—quantification of IR cells was done using Neurolucida 2020 and Neurolucida Explorer (MBF Bioscience, Williston, Vermont, USA) on confocal images of immunofluorescent histological slides.

From each species, three brain specimens were used for quantification. Slides were analyzed from each STN region (anterior, central, or posterior STN) and each double‐labeling combination (nNOS + FOXP1, HuC/HuD + FOXP1, and HuC/HuD + nNOS). Coordinates of STN regions (anterior, central, posterior) are defined as described in Table 3.

**TABLE 3:** The defined antero‐posterior stereotactic range for each STN region in the three species according to the stereotactic atlases.

Species	Anterior STN	Central STN	Posterior STN	References
Mouse	From −1.70 mm to −1.82 mm	From −1.94 mm to −2.06 mm	From −2.18 mm to −2,30 mm	Paxinos and Franklin (2004)
Rat	From −3.12 mm to −3.36 mm	From −3.48 mm to −3.72 mm	From −3.84 mm to −4.08 mm	Paxinos and Watson (2007)
Human	From 12.0 mm to 13.3 mm	From 14.6 mm to 16.0 mm	From 17.2 mm to 18.6 mm	Mai et al. (2004)

During analysis, the region of interest was delimited as the entire STN on a coronal section plane, and all IR neurons were marked and counted—digital labels were placed layered on top of signals from immunolabeled cells. Traced images were then split from original images and exported as data files and vector graphics files to allow further analysis.

The results of the quantification are presented as the proportion of FOXP1+ and nNOS+ neurons within the total STN neuron population (defined as the total number of HuC/HuD+ cells). Figures were edited and assembled in ImageJ and Adobe Photoshop.

## 3 Results

In this study, the expression patterns of FOXP1 and nNOS were analyzed in the adult mouse, rat, and human STN, along with their proportions within the estimated total population of STN neurons. We performed immunofluorescent double‐labeling combining selected markers, FOXP1 and nNOS, with each other, but also with a cytoplasmic pan‐neuronal marker (HuC/HuD) and a nuclear marker DAPI. Signals from HuC/HuD and nNOS can be found in the cytoplasm, while signals from DAPI and FOXP1 can be found in the nucleus (Figures 1, 2, 3). In addition, we calculated the density of nNOS+, FOXP1+, and Huc/HuD+ neurons in the STN. The largest density of STN neurons was present in the mouse STN, followed by rat and human STN (Table 4).

**FIGURE 1:**
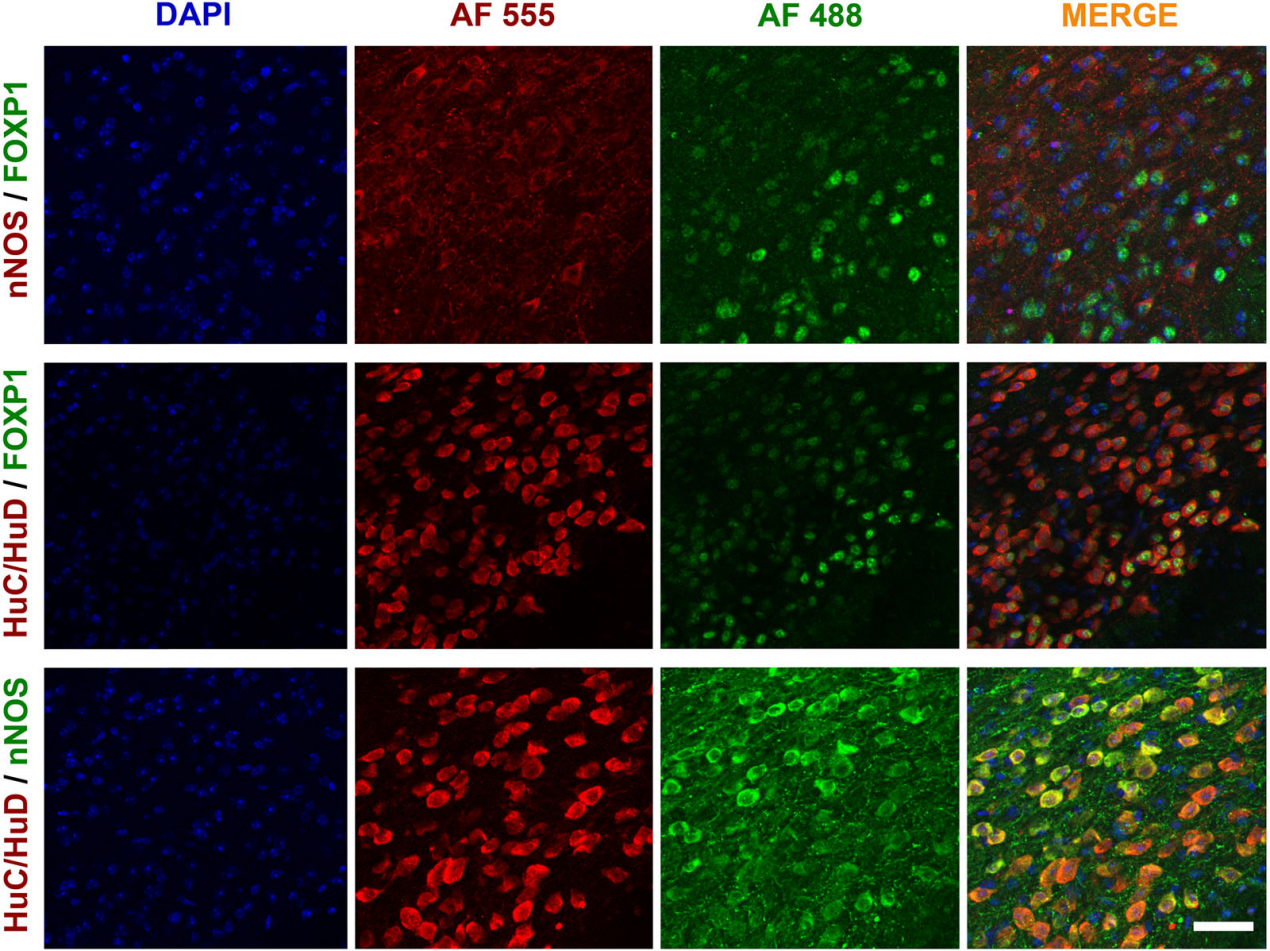
Immunofluorescent double‐labeling of neurons in the mouse STN with different combinations of primary antibodies. First row: mouse anti‐nNOS (red) + rabbit anti‐FOXP1 (green); second row: mouse anti‐HuC/HuD (red) + rabbit anti‐FOXP1 (green); third row: mouse anti‐HuC/HuD (red) + rabbit nNOS (green). Nuclei are stained with DAPI (blue). Secondary antibodies are donkey anti‐mouse AlexaFluor 555 (red), donkey anti‐rabbit AlexaFluor 488 (green). The scale bar represents 50 µm.

**FIGURE 2:**
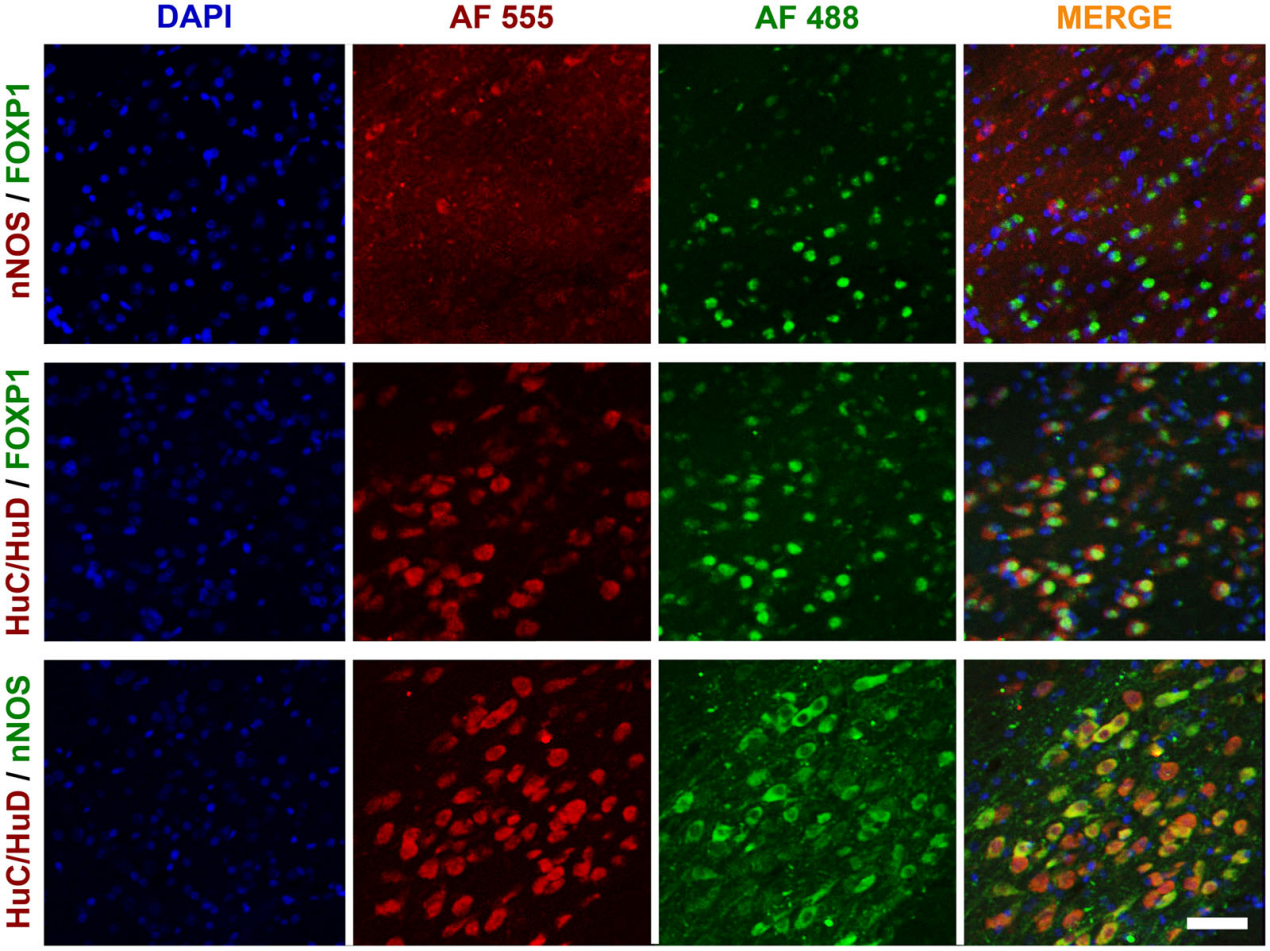
Immunofluorescent double‐labeling of neurons in the rat STN with different combinations of primary antibodies. First row: mouse anti‐nNOS (red) + rabbit anti‐FOXP1 (green); second row: mouse anti‐HuC/HuD (red) + rabbit anti‐FOXP1 (green); third row: mouse anti‐HuC/HuD (red) + rabbit nNOS (green). Nuclei are stained with DAPI (blue). Secondary antibodies are donkey anti‐mouse AlexaFluor 555 (red), donkey anti‐rabbit AlexaFluor 488 (green). The scale bar represents 50 µm.

**FIGURE 3:**
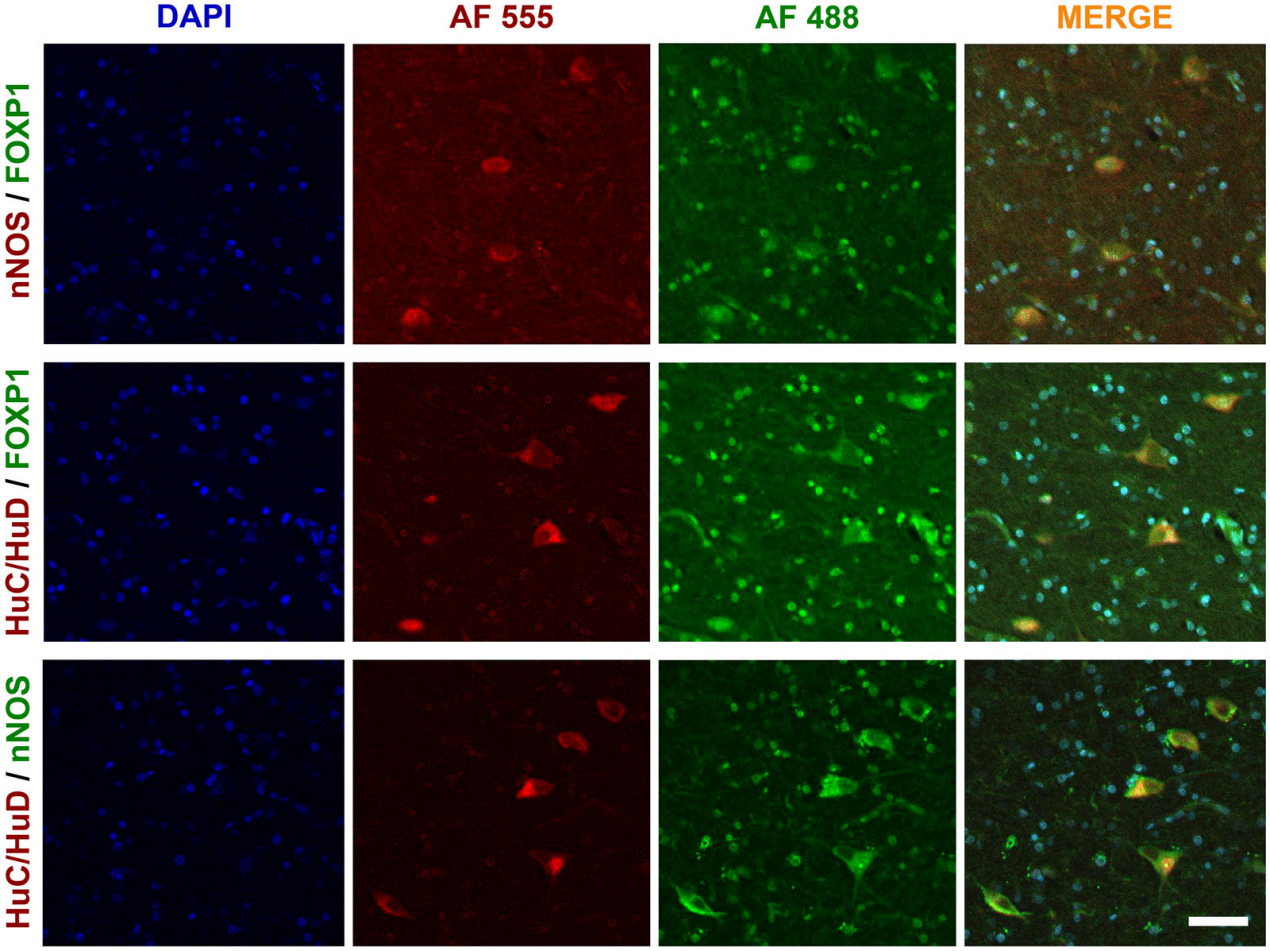
Immunofluorescent double‐labeling of neurons in the human STN with different combinations of primary antibodies. First row: mouse anti‐nNOS (red) + rabbit anti‐FOXP1 (green); second row: mouse anti‐HuC/HuD (red) + rabbit anti‐FOXP1 (green); third row: mouse anti‐HuC/HuD (red) + rabbit nNOS (green). Nuclei are stained with DAPI (blue). Secondary antibodies are donkey anti‐mouse AlexaFluor 555 (red), donkey anti‐rabbit AlexaFluor 488 (green). The scale bar represents 50 µm.

**TABLE 4:** The density of nNOS, FOXP1, and HuC/HuD neurons in the mouse, rat, and human STN.

	Mouse	Rat	Human
**HuC/HuD**	150,016.7 ± 40,965.9	67,882.6 ± 18,289.2	4063.2 ± 2889.2
**FOXP1**	67,337.1 ± 44,916.9	35,098.5 ± 15,344.7	1442.7 ± 394.3
**nNOS**	45,157.3 ± 21,853.6	27,911.0 ± 7958.8	3015.6 ± 1886.8

Reported values are mean ± SD neurons/mm^3^.

### 3.1 FOXP1 and nNOS Are Expressed in Distinct Zones in the Rodent STN but Not in the Human STN

Immunofluorescently stained coronal STN sections reveal a distinct spatial distribution of FOXP1+ and nNOS+ neuronal populations across three different regions and three different species. The images show that the expression of FOXP1 and nNOS is spatially differentiated in rodents (Figures 4 and 5), separated along the dorsoventral axis.

**FIGURE 4:**
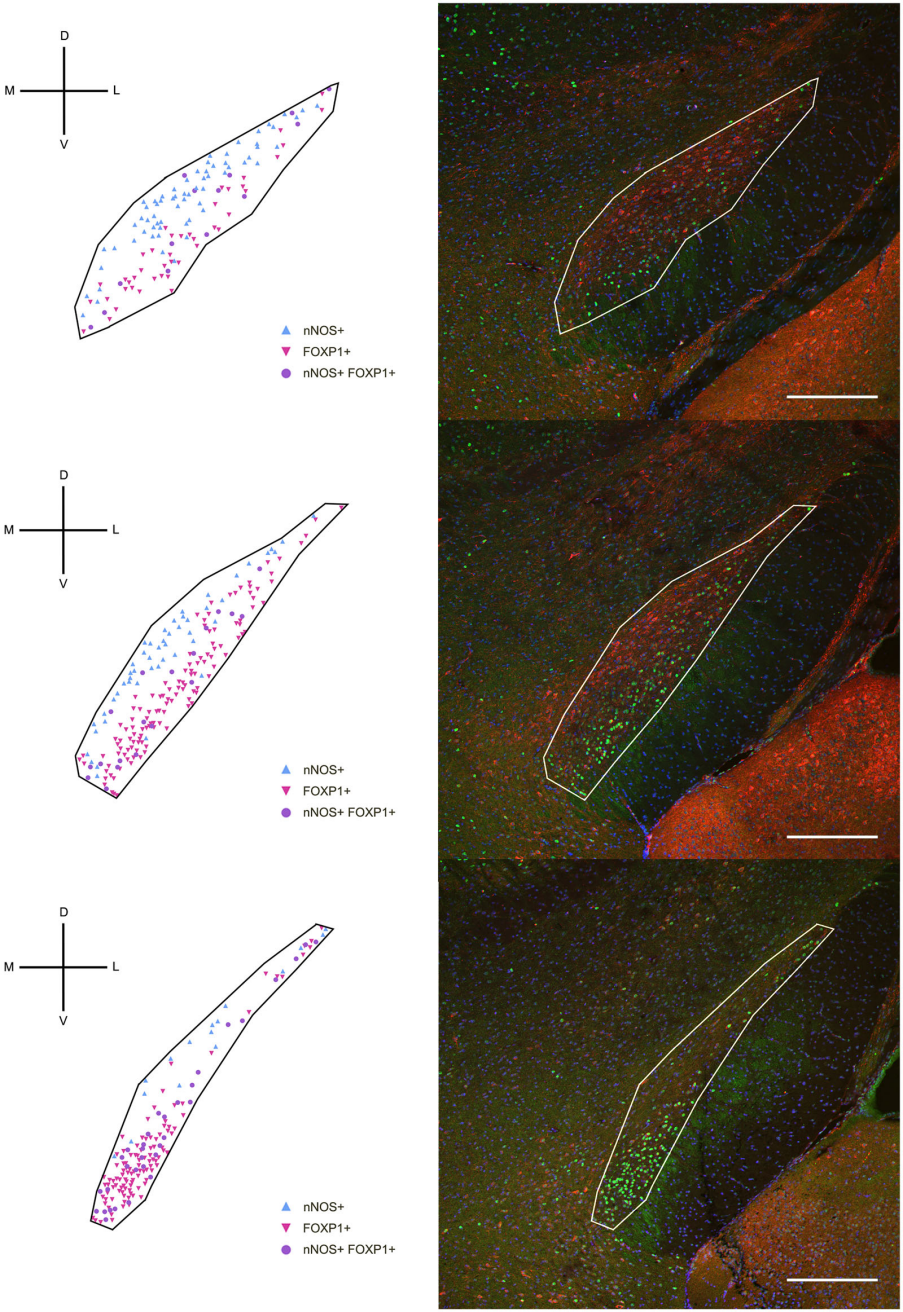
Image tracings of immunofluorescent mouse coronal slides from the anterior (first row), central (middle row), and posterior (bottom row) STN, with immunoreactive neurons labeled: nNOS+ cells are marked with blue symbols, FOXP1+ cells are marked with pink symbols, and nNOS+/FOXP1+ cells are labeled with purple symbols. White scale bars in each tracing represent the length of 250 µm.

**FIGURE 5:**
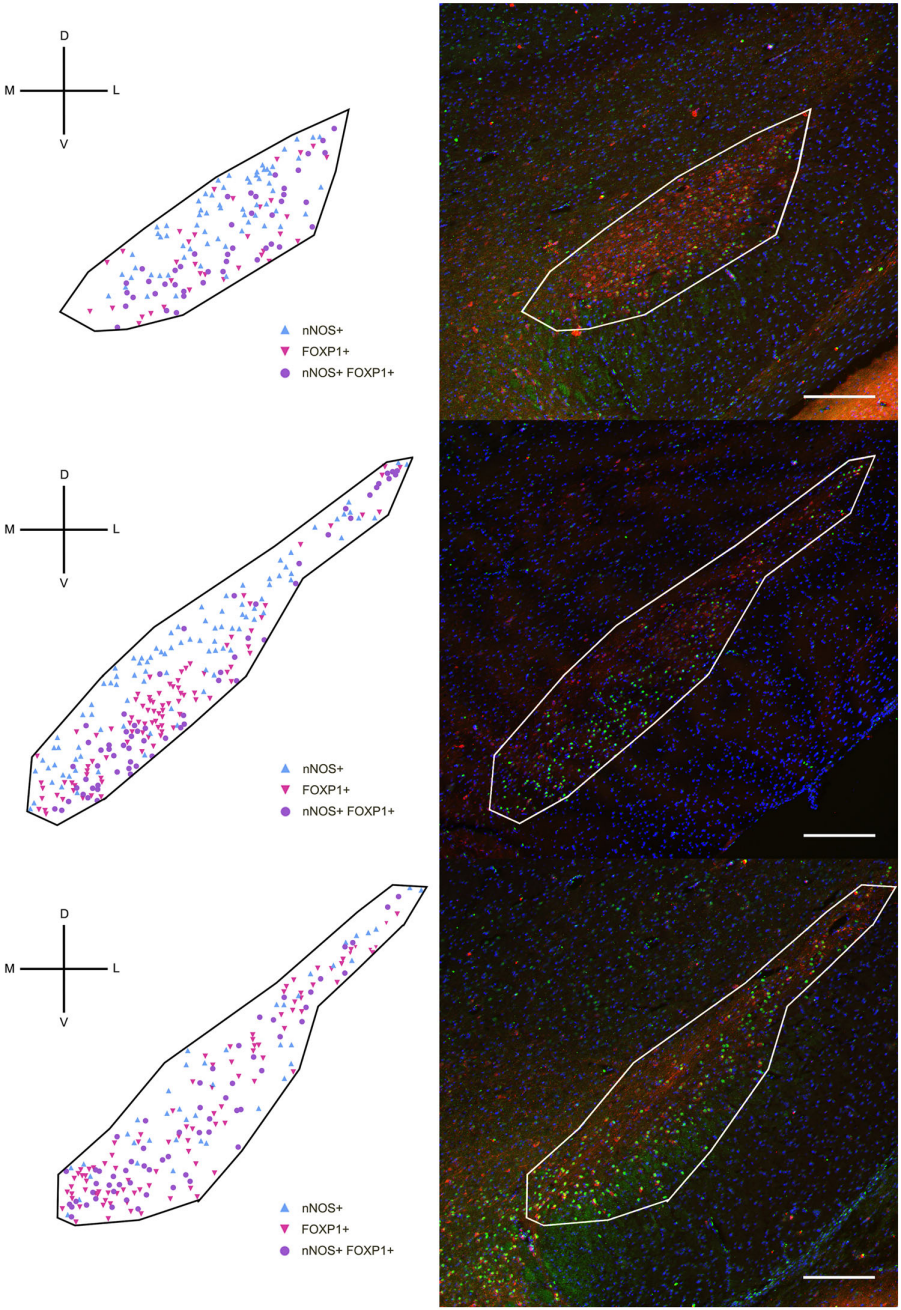
Image tracings of immunofluorescent rat coronal slides from the anterior (first row), central (middle row), and posterior (bottom row) STN, with immunoreactive neurons labeled: nNOS+ cells are marked with blue symbols, FOXP1+ cells are marked with pink symbols, and nNOS+/FOXP1+ cells are labeled with purple symbols. White scale bars in each tracing represent the length of 250 µm.

The FOXP1 and nNOS populations are clearly separated and almost nonoverlapping in the mouse STN. FOXP1 neuron localization is restricted to the ventrolateral portion of STN, while nNOS neurons are restricted to the dorsomedial portion. Neurons coexpressing both FOXP1 and nNOS represent a small fraction of neurons in the mouse STN and are usually located within the ventrolateral portion containing predominantly FOXP1+ neurons.

In rats, the neuronal populations exhibit similar expression patterns to those observed in the mouse STN, with some differences. The FOXP1+ population is still restricted to the ventrolateral STN, while the nNOS+ population is predominant in the dorsolateral STN. However, the nNOS expression is extended outside of the dorsolateral STN to the ventral part of the STN, and a part of FOXP1+ neurons there coexpress nNOS. In general, contrary to the pattern in the mouse STN, there are some interspersed nNOS+ neurons within the FOXP1+ population. This interspersion is especially evident in the posterior part of the STN, giving it a nearly uniform appearance (as opposed to two distinct populations). The FOXP1+/nNOS+ population is greater in rat STN compared to mouse STN, but most of these neurons can still be found in the ventromedial zone predominantly occupied by FOXP1+ neurons (Figure 5).

In the human STN, these neuronal populations do not exhibit spatially restricted expression patterns (Figure 6). Both FOXP1+ and nNOS+ neurons are found to be intermixed and dispersed evenly throughout the nucleus, with substantial colocalization. FOXP1+/nNOS+ neurons are a large subpopulation in the human STN, and similar to the FOXP1+ and nNOS+ neurons, are also dispersed throughout the entire STN. Taken together, this shows that while the expression of FOXP1 and nNOS is present in STN neurons of all species, it is differently spatially organized. Human STN exhibits a more homogenous distribution of neuronal populations, while rodent STN neuronal populations tend to spatially segregate.

**FIGURE 6:**
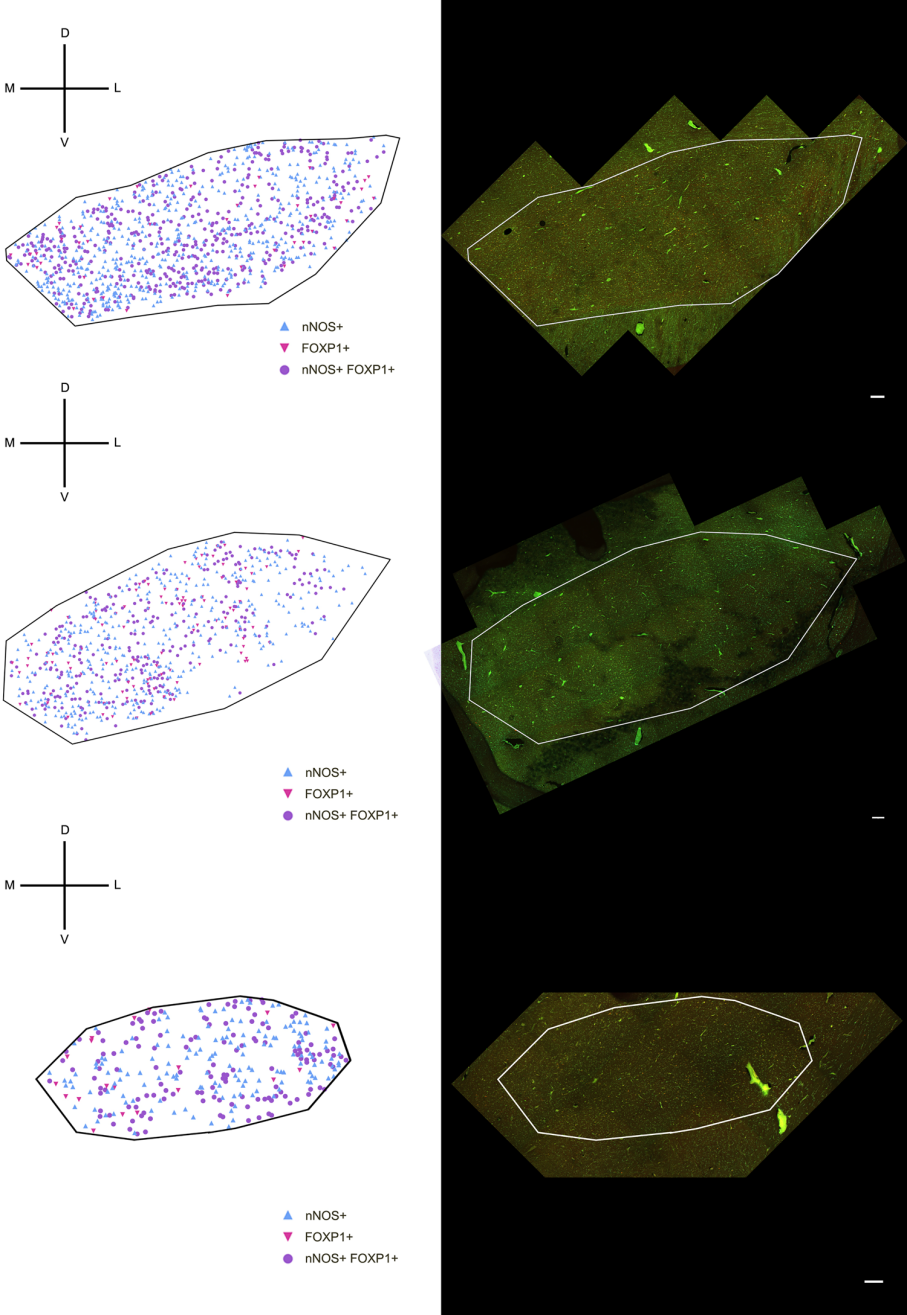
Image tracings of immunofluorescent human coronal slides from the anterior (first row), central (middle row), and posterior (bottom row) STN, with immunoreactive neurons labeled: nNOS+ cells are marked with blue symbols, FOXP1+ cells are marked with pink symbols, and nNOS+/FOXP1+ cells are labeled with purple symbols. White scale bars in each tracing represent the length of 250 µm.

### 3.2 Human STN Neurons Exhibit Significant Expansion of the nNOS+ Neuronal Subpopulation

Qualitative analysis indicated that there are significant differences in the number of FOXP1+, nNOS+, and FOXP1+/nNOS+ neurons in the STN between the analyzed species. Therefore, we set out to quantify the number of neurons expressing each marker and the number of neurons expressing both markers. For each molecular marker, we quantified the number of IR cells in three STN regions (anterior, central, and posterior) in all three species (mouse, rat, and human). The total number of neurons in the STN slices was calculated as the total number of HuC/HuD+ cells. From colocalizations of HuC/HuD with FOXP1 and nNOS, proportions of FOXP1+ and nNOS+ populations in HuC/HuD+ cells were calculated.

When the number of counted IR cells is averaged across slices for each species (Figure 7), FOXP1+ neurons account for 40%–45% of all STN neurons in all three species (41.2% in mice, 46.5% in rats, and 41.4% in humans). Percentages of nNOS+ neurons show higher interspecies variation, spanning from 31.9% in mice, 42.6% in rats, to 67.8% in humans. Given that the FOXP1+ neuron shares are quite consistent across species, the rise in nNOS+ neuron proportions across species is followed by a consequential rise in the FOXP1+/nNOS+ colocalization. Populations of FOXP1+/nNOS+ neurons make up, on average, 6.7% of all neurons in mice, 19.5% of all neurons in rats, and 30.3% of all neurons in humans. The percent of FOXP1−/nNOS+ neurons is the smallest in mice (25.2%) and largest in humans (37.5%), while, inversely, the percent of FOXP1+/nNOS− neurons is the largest in mice (34.5%) and smallest in humans (11.1%).

**FIGURE 7:**
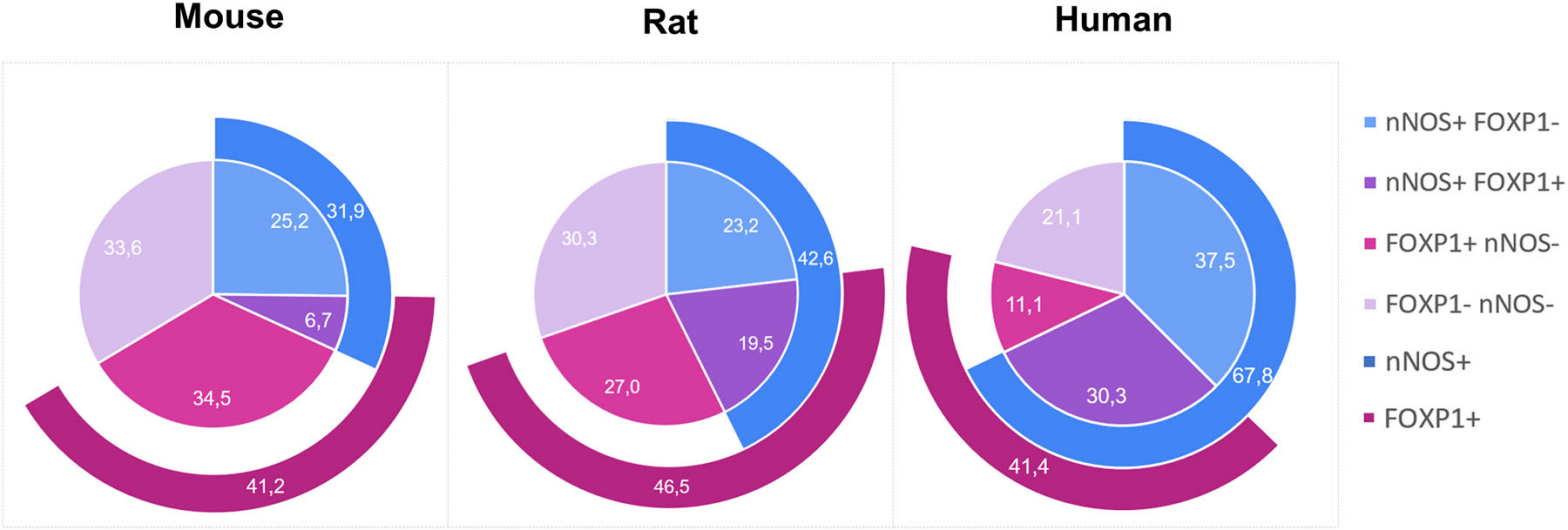
Images show average shares of neuronal populations expressing nNOS and FOXP1 in a general neuronal population in the STN, for mouse, rat, and human, respectively.

### 3.3 Both FOXP1 and nNOS Exhibit Anterior–Posterior Expression Gradients Within the STN

Next, we set out to see if observed changes in the STN's neuronal subpopulation are global or can be attributed to a specific part of the STN. We grouped the cell counts based on the antero‐posterior location of the slices into anterior STN, central STN, and posterior STN (Figure 8) to make observations on the spatial distribution of neurons across STN and across species. Both FOXP1 and nNOS showed regional variations in the percentage of positive neurons. There is a significant variation in FOXP1+ neuron regional abundance and distribution within the STN, and each species exhibits a specific antero‐posterior expression pattern of FOXP1 in the STN. The proportion of FOXP1+ neurons in rodents is the highest in the posterior sections of the STN. Inversely, the proportion of FOXP1+ neurons in humans is the highest in the anterior STN slices.

**FIGURE 8:**
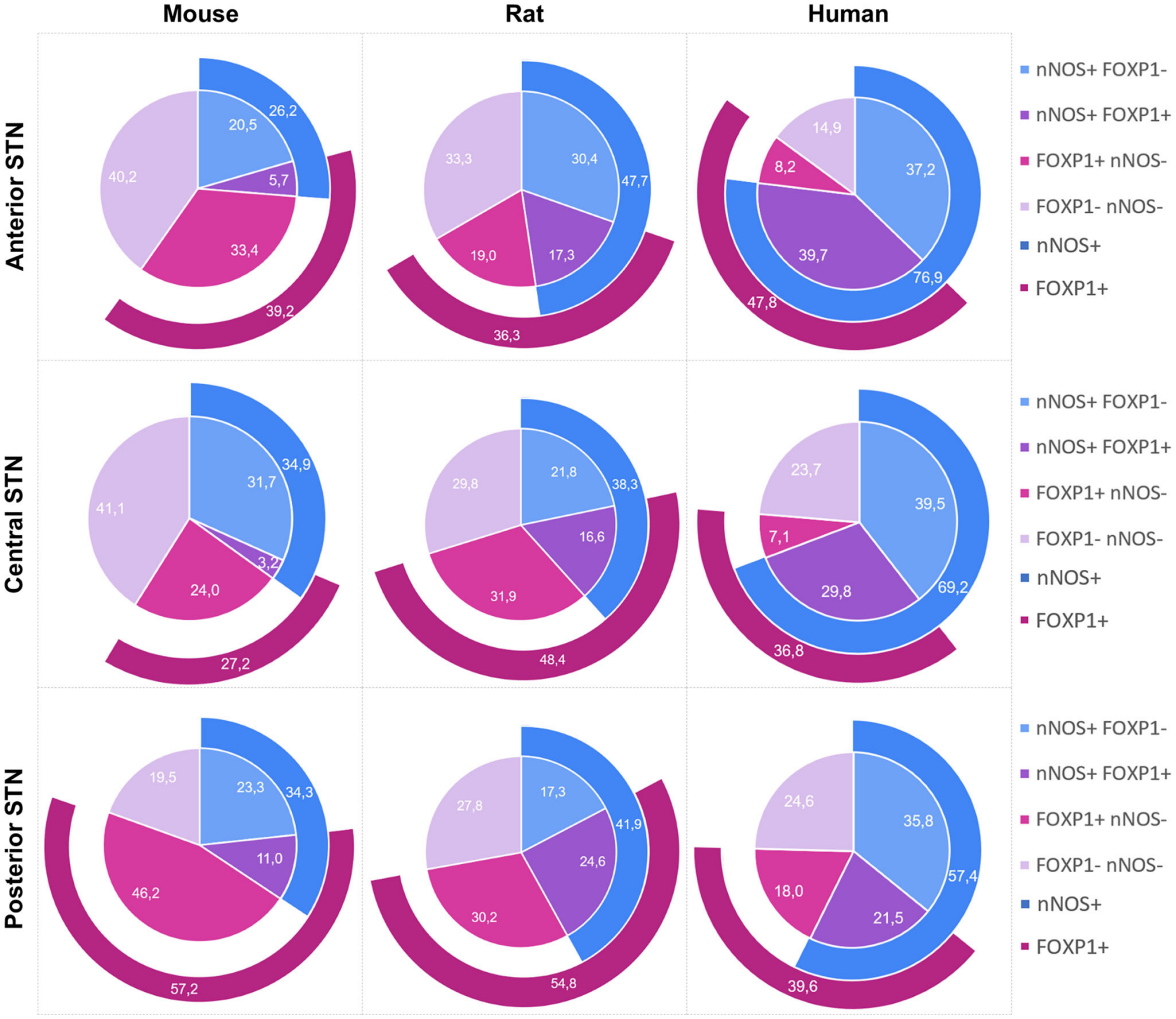
Images show average shares of neuronal populations expressing nNOS and FOXP1 across different STN regions (anterior, central, posterior) for mouse, rat, and human STN, respectively.

In mice, the largest percentage of FOXP1+ neurons can be observed in the posterior part (57.2%) followed by the anterior (39.2%) and the central (27.2%). In the rat STN, an increase in the percentage of FOXP1+ neurons from anterior to posterior can be observed (anterior—36.3%, central—48.4%, posterior—54.8%; Figure 8). In human STN the largest percentage of FOXP1+ neurons can be observed in the anterior part (47.8%) while central and posterior have roughly the same percentage of FOXP1+ neurons (36.8% and 39.6%, respectively; Figure 8).

The smallest share of nNOS+ neurons in mice can be found in the anterior STN (26.2%), while central and posterior part contained approximately the same percentage of nNOS+ neurons which was approximately a third of all STN neurons (34.9% and 34.3%, respectively; Figure 8). The expression pattern was reversed for the rat, with the highest percentage of nNOS+ neurons in the anterior part (47.7%) while central and posterior parts showed roughly the same percentage of nNOS+ neurons (38.3% and 41.9%, respectively; Figure 8). In human STN, we observed an antero‐posterior gradient of nNOS+ neurons. The highest percentage of nNOS+ neurons was recorded in the anterior part (76.9%), followed by the central (69.2%) and the posterior (57.4%) part (Figure 8).

Consequently, the changes in the proportions of each analyzed marker also led to the changes in the percentage of FOXP1+/nNOS+ subpopulation. It is interesting to note that although the percentage of FOXP1+/nNOS+ neurons increased in rats, the general expression pattern is the same as in mice. In both mice and rats, the largest percentage of FOXP1+/nNOS+ neurons was observed in the posterior part, while the anterior and central parts had roughly the same percentage of these neurons. However, in the human STN, the largest percentage of FOXP1+/nNOS+ neurons was observed in the anterior part (39.7%), followed by the central (29.8%) and the posterior (21.5%) part (Figure 8).

In the anterior part of the human STN, an increase in the percentage of FOXP1+, nNOS+, and FOXP1+/nNOS+ neurons can be observed, compared to rodents. As a result, the number of neurons which are FOXP1−/nNOS− (only HuC/HuD+) is the smallest in the anterior part (14.9%) while the percentage of these neurons in the central and posterior parts roughly corresponds to the percentages observed in rodents (Figure 8).

Overall, these data suggest that the increase in the nNOS+ neuronal population within the human STN is mainly due to growth in the anterior and central regions. This expansion results from the expression of nNOS in neurons previously negative for FOXP1 and nNOS, as well as from the expression of nNOS in a subset of FOXP1+ neurons.

## 4 Discussion

In this study, we reveal expression patterns of FOXP1 and nNOS proteins in the adult STN of three species: human, mouse, and rat. Results of this study provided us with several novel findings: (i) the data presented here provide first evidence of FOXP1 protein expression in the adult rat and human STN, while also confirming it in the mouse STN; (ii) FOXP1+ and nNOS+ neuronal subpopulations are spatially segregated in the rodent STN while intermixed and homogeneously distributed through the entire human STN; and (iii) there is a noticeable expansion of nNOS+ and FOXP1+/nNOS+ neuronal subpopulations in the human STN compared to the rodent STN.

### 4.1 FOXP1‐Expressing Neurons Are a Large Neuronal Subpopulation in the Adult Mammalian STN

FOXP1 is an important factor in the development of the motor system (Dasen et al. 2008; Palmesino et al. 2010; Chasse et al. 2024). Here, we demonstrate that FOXP1+ neurons are a large (approximately 40%) neuronal subpopulation in adult STN of mice, rats, and humans. Our findings confirm previous findings in adult mouse STN (Skidmore et al. 2008), extend findings of fetal FOXP1 expression in the human STN (Bokulić et al. 2025), and provide the first evidence of FOXP1 presence in rat STN.

The functional role of FOXP1 in the STN is still unknown. However, conditional knockout of the *Foxp1* gene in mice, as well as mutations in the human FOXP1, result in a wide array of motor, cognitive, and behavioral disorders (Araujo et al. 2015; Bacon et al. 2015; Meerschaut et al. 2017; Siper et al. 2017; Co et al. 2020; Lozano et al. 2021). Many of the mentioned symptoms are also present in various disorders of the STN (Adey et al. 1962; Baunez and Robbins 1997; Baunez et al. 2007; Brittain et al. 2012; Choi et al. 2018; Chudasama et al. 2003; Frank et al. 2007; Lindsley et al. 1970; Mallet et al. 2008; Papuć 2015; Parolari et al. 2021; Polosan et al. 2019; Sensi et al. 2004; Voon et al. 2017).

Furthermore, a recent retrograde tracing study by Kondabolu et al. (2023) showed that the mouse STN projects to parvalbumin‐expressing interneurons of the striatum. Retrogradely labeled neurons in the STN are predominantly located on the medial‐ventrolateral portion of the STN, which spatially corresponds to the mouse STN FOXP1+ population in our study. Based on these reports and our findings, we argue that FOXP1 may be an important factor in the normal functioning of the STN and that some of the observed symptoms in the disruptions of the FOXP1 are due to its role in the STN. However, to fully understand the role of FOXP1 in the STN, additional studies which will focus exclusively on the FOXP1 in the STN are needed.

### 4.2 Nitrinergic Neurons in the Adult Mammalian Subthalamic Nucleus

Nitrinergic neurons are one of the largest subpopulations in the STN, as previously reported by multiple papers (Vincent and Kimura 1992; Endoh et al. 1994; Rodrigo et al. 1994; Nisbet et al. 1994; Eve et al. 1998; De Vente et al. 1998; Cha et al. 2000; Cavalcanti‐Kwiatkoski et al. 2010; Czarnecka et al. 2013; Santos‐Lobato et al. 2016; Bokulić et al. 2021; Parolari et al. 2021). In human STN, nNOS+ neurons were reported to constitute 90%–95% of all neurons (Nisbet et al. 1994; Eve et al. 1998; Santos‐Lobato et al. 2016; Bokulić et al. 2021), while data on the expression of the nNOS in the rodent STN are somewhat limited (Vincent and Kimura 1992; Endoh et al. 1994; Rodrigo et al. 1994; De Vente et al. 1998; Cha et al. 2000; Czarnecka et al. 2013; Parolari et al. 2021). This study also confirms clear nNOS expression in the STN of all three species. Proportions of nNOS+ neurons are more variable across species in comparison to FOXP1 expression. On average, nNOS+ neurons constitute ∼32% of all neurons in the mouse STN, ∼43% in the rat STN, and ∼68% in the human STN.

It is interesting to note that nNOS+ neuronal subpopulation is significantly expanded in the human STN compared to the rodents. While FOXP1+ neuronal subpopulation is relatively stable in relative size in all three species (approximately 40%), the nNOS+ subpopulation spanned from 30% in mice to approximately 70% in humans. To date, the exact role of nNOS in the functioning of the STN is not known. One study analyzing the expression of nNOS mRNA in basal ganglia of patients with Parkinson's disease found the increase of nNOS mRNA expression in the STN, albeit without clear functional implications (Eve et al. 1998).

An electrophysiological study by Sardo et al. (2006) recorded changes in the discharge of STN neurons in the rat after inhibiting the nitric oxide synthase with two different inhibitors. They found that eight out of 10 cells respond to 7‐NI with a firing rate decrease, while two cells showed no response. Out of 32 cells treated with L‐NAME, 18 exhibited decreased firing rate (∼56%), six neurons showed an increase in the firing rate (∼19%), and eight were not responsive (25%). It is interesting to note that the most of the inhibited cells tend to be in the dorsal part of the STN (Sardo et al. 2006). Furthermore, in Parolari et al. (2021), *Nos1* mRNA seems to be accumulated mainly dorsally even though this was not explicitly stated by the authors, but it can be seen in the presented figures. In these two studies, the nNOS+ neurons are in the same region as in our study.

In general, nitric oxide signaling in the brain is involved in neuroplasticity, neurotransmitter modulation, dopamine signaling, and aging (West et al. 2002; Tricoire and Vitalis 2012; Chachlaki and Prevot 2019). Due to the sheer number of nitrinergic neurons in the human STN, it would be interesting to see what the exact role of nNOS and/or NO is in the physiology of the STN.

### 4.3 Differences in the STN Neuronal Proportions and Densities Between Studies

The obtained proportion of nNOS neurons in the human STN somewhat differs from the existing literature—a study by Santos‐Lobato et al. (2016) estimated that about 90% of all STN neurons express the nNOS protein. This difference could be explained by a few methodological differences. Santos‐Lobato et al. (2016) used Nissl staining to label all neurons in the STN and estimate the proportion of nNOS neurons counted on immunohistochemically stained sections. We used HuC/HuD as a pan‐neuronal marker to label all neurons through immunofluorescent double‐labeling, where colocalization is directly evident on the same section and the same cell bodies. Furthermore, Santos‐Lobato et al. (2016)used a square grid to count neurons in every second square and estimate the total neuron count. This can introduce additional variation in the obtained estimate values, so we counted the total STN area on coronal sections. Additionally, they used a different primary anti‐nNOS antibody on human tissue with different sample sizes, postmortem intervals (PMIs), and fixation times. It is possible that these differences in tissue processing, antibody properties, as well as blocking and antigen retrieval techniques, could all accumulate and contribute to the difference in the staining density between studies, and subsequent neuronal population estimates.

In our previous study, we already reported that both Nissl and NeuN are not good “pan‐neuronal” markers in the STN due to technical limitations (Bokulić et al. 2021). Namely, NeuN, when used on formalin‐fixed paraffin‐embedded tissue, does not stain all neurons (Luijerink et al. 2021), and to declare a cell as a neuron on Nissl stain, a stringent criterion needs to be followed, which could lead to underrepresentation of neurons (Bokulić et al. 2021). Thus, it is possible that previous studies underreported the number of STN neurons. Therefore, we decided to use a novel “pan‐neuronal” marker HuC/HuD to label all neurons.

The neuronal density in our study is larger than that reported in studies using either Nissl or NeuN as pan‐neuronal markers (Lange et al. 1976; Oorschot 1996; Hardman et al. 2002; Bokulić et al. 2021). The one study that also used HuC/HuD reported values similar to our own (Zwirner et al. 2017). Furthermore, the density of nNOS neurons in the STN in this study and our previous study is comparable. We also used fluorescent double‐labeling immunohistochemistry, which labeled both HuC/HuD and nNOS in the same neuron, allowing us to determine the total number and percentages of nNOS‐ir neurons in the same sample. Therefore, due to the larger number of total STN neurons, the percentage of nNOS‐positive neurons in this study is smaller, although the density is similar to previously reported values.

### 4.4 Implications for STN Subdivision Into Anatomical and Functional Domains Across Species

A widespread postulation regarding STN in a clinical context is that STN consists of three anatomically and functionally distinct domains—a dorsolateral motor region, ventromedial associative region, and the medial limbic tip. This tripartite theory has been described in many STN review articles (DeLong et al. 1985; Parent and Hazrati 1995; Joel and Weiner 1997; Hamani et al. 2004; Romanelli et al. 2005; Temel et al. 2005; Bevan et al. 2006; Benarroch 2008) and is, at least on a functional level, supported by a few electrophysiological recordings in humans (Mallet et al. 2008; Greenhouse et al. 2011). However, there have been many attempts at establishing meaningful subdivisions of the STN, especially in primates (including humans), based on anatomy, connectivity, and cytoarchitectonics. This has been covered in detail by Keuken et al. (2012), who summarized 33 original studies and showed that the number of described compartments of the primate STN in these studies ranges from 0 to 4. Based on this variability, they concluded that the tripartite model of the primate STN, dominant in STN reviews, is not in fact supported by original published articles.

A recent report by Miletić et al. (2022), using fMRI on healthy volunteers, could not find functional subdivisions within the STN. On the other hand, Bertrand et al. (2024) demonstrated using in vivo STN stimulation, in rhesus monkeys, that various functions of the STN can be elicited from the entire STN, although motor responses are more easily elicited by stimulating the dorsolateral part and cognitive responses are elicited by stimulating the ventromedial part. They further argue that some functional segregation exists, but with extensive overlap (Bertrand et al. 2024). Molecular marker expression patterns, such as calcium binding proteins or glutamatergic and GABAergic markers provide no evidence for anatomical borders with clear subdivisions in the human STN, but rather follow either nonlinear expression patterns across space, or linear expression gradients across the dorsolateral–ventromedial axis (Augood et al. 1999; Levesque and Parent 2005; Alkemade et al. 2019).

In this study, we found that FOXP1 and nNOS neuronal populations in human STN were highly colocalized, intermixed, and homogeneously distributed. We found a slight nNOS+ decreasing density gradient along the antero‐posterior axis, which is followed by a change in the rate of FOXP1/nNOS colocalization, given that the FOXP1 distribution density is fixed along the same axis. Other than that, we found no evident spatial segregation of FOXP1 and nNOS neuronal populations, and subsequently, no evidence of any kind of STN subdivision in humans solely based on these markers. There is an abundance of papers on STN subdivisions in primates with many different viewpoints, and results from our study do not support the existence of three distinct domains in the human STN, but rather support the argument that the tripartite model should be revised (Alkemade and Forstmann 2014; Keuken et al. 2012).

As opposed to our results on human STN, we found that the FOXP1+ and nNOS+ populations tend to be spatially segregated in the rodent STN. Whereas no domains could be identified in the human STN, the rodent STN could be divided into two domains based on FOXP1+ and nNOS+ distribution: a dorsomedial STN, abundant with nNOS+ population; and a ventrolateral STN, abundant with FOXP1+ population. In comparison with studies on primates, there are not as many published papers attempting to divide the rodent STN into anatomo‐functional domains. Studies on mice are, due to animal model characteristics, mostly focused on transcriptomics and optogenetics.

A paper by Wallen‐Mackenzie et al. (2020) proposed spatial domains in the mouse STN based on their data obtained by RNA sequencing of PITX2+ neurons: STNa (ventromedial STN expressing Col24a1, Calb2, Nxph4), STNb (central STN expressing Pvalb, Col24a, Col24a, Nxph4), STNc (dorsolateral STN expressing Pvalb and Nxph4), and STNd (dorsal strip of the STN expressing Gad; Wallen‐Mackenzie et al. 2020; Prasad and Wallen Mackenzie 2024). A study by Parolari et al. (2021) attempted to find distinct neuronal populations in the mouse STN with in situ hybridization to map mRNA expression. They used Pitx2 as a pan‐neuronal marker in the STN colabeled with Nos1, Ndnf, and Gabrr3. They found that Nos1+, Ndnf+, and Gabrr3+ cells represent 55%, 70%, and 65% of Pitx2+ STN neurons. It is important to note that cell profiling in both of these studies is based on mRNA expression, which may not be a reflection of the ultimate functional proteomic profile due to both post‐transcriptional and post‐translational regulation of gene expression. However, in Parolari et al. (2021), Nos1 mRNA seems to be accumulated mainly dorsally, consistent with protein expression in our findings, even though this was not explicitly stated by the authors, but it can be seen in the presented figures.

Meanwhile, studies on rats are mainly focused on electrophysiology and connectivity. Lardeux et al. (2009). used electrophysiological recordings to study distinct neuronal populations in the rat STN. They showed that different populations of STN neurons in rats can differentiate between different reward categories and selectively encode sucrose or cocaine (Lardeux et al. 2013), but also between reward quantity within the same category—some STN neurons (∼44%) in rats respond to cues for 4% sucrose, some (∼43) respond to cues 32% sucrose solution while ∼14% show similar response to both cues (Lardeux et al. 2009). Unfortunately, the spatial distribution of these neuronal populations in the rat STN was not described in the papers, so it would be interesting to investigate whether they could be grouped along the dorsoventral axis.

Groenewegen and Berendse (1990) used tracing to investigate the connections of the STN in the rat. They found that “the dorsomedial part of the STN is connected with the subcommissural ventral pallidum, whereas a more ventral and lateral part of the medial STN is related to the medial globus pallidus.” They also showed that the STN is innervated by the medial prefrontal cortex (mPFC; Berendse and Groenewegen 1991). Based on data from the previous literature, their report, and unpublished observations, they defined the ventrolateral portion of the medial STN as a connection point receiving projections from the dorsal anterior cingulate cortex (dACC) and the mPFC (Berendse and Groenewegen 1991). Kita and Kitai (1987) found that STN projections in the rat display a dorsoventral topography—dorsal STN sends projections to the ventral GP and dorsal SNR, while the ventral STN sends projections to the dorsal GP and ventral SNR.

Compared to our results, where we found the dorsomedial STN to be nNOS+ populated and ventrolateral STN to be FOXP1+ populated, we can suspect that nNOS in the rodent STN marks neurons connecting to the ventral pallidum, while FOXP1 is characteristic for neurons connecting to the dorsomedial globus pallidus as well as striatal parvalbumin‐expressing interneurons. The FOXP1‐expressing neurons would also encompass the portion of the STN receiving projections from the mPFC and dACC. As there is a certain degree of overlap in the rodent subthalamic FOXP1 and nNOS populations, ∼7% average of colocalization in the mouse STN and ∼20% average in the rat STN, it would be interesting to find what functional difference the FOXP1+ nNOS+ neurons bear.

In this study, based on FOXP1+ and nNOS+ neuron distributions, rodent STN appears to have a defined internal organization with segregated neuronal populations forming visibly distinct subdomains, whereas the human STN appears to have complex organization with diffusely intermixed populations of neurons. These organizational interspecies differences are not confined to the STN—other subcortical structures show similar characteristics with rodent structures being more defined. The most well‐known example is the hypothalamus—the nuclear masses in the human hypothalamus are rarely as well differentiated as in other species and their ambiguous outlines show great variability in different individuals (Saper 2012; Dudas 2021). While the topography of the mouse hypothalamus, with its well‐defined nuclei, is described in greater detail, human hypothalamus is characterized by cytoarchitectonic indistinctness where most of the structures described as nuclei are actually areas without distinct boundaries (Saper 2012; Dudas 2021). Since the STN, based on its development, does appear to be of hypothalamic origin (Barbier and Risold 2021; Bokulić et al. 2025), it is not surprising to find common themes in their organizational patterns between species.

### 4.5 Limitations of the Study

The main methodological limitation of this study is a significant difference in the tissue quality of rodent and human samples. Rodent samples are superior in quality with no PMI, while human samples deteriorate in quality with high PMI in comparison to rodent samples. PMIs of the human samples introduce variability in the obtained results and complicate comparisons between humans and rodents, as well as comparisons with previous human studies.

## 5 Conclusion

In conclusion, this study provides the first evidence of FOXP1 protein expression in the adult rat and human STN. This paper also confirms FOXP1 expression in the mouse STN and a clear nNOS expression in the STN of all species. Results reveal interspecies differences in the spatial patterning of nNOS‐ and FOXP1‐expressing neurons, where rodent STN shows dorsoventral patterning which divides it into two domains, while human STN shows a homogeneous distribution of intermixed neuronal populations without any clear subdomains. Nonetheless, clear division of neuronal populations is shown in the rodent STN, and future research should aim to investigate further characterization and functional significance of these populations. Since results indicate that cytoarchitectonic segregational organization of the human/primate STN is much less strict than in rodents, this warrants caution when translating clinically relevant data from rodents onto primates.

## Author Contributions

Conceptualization was done by Tila Medenica and Goran Sedmak. Tila Medenica and Ema Bokulić performed the experiments. Tila Medenica and Goran Sedmak wrote the first draft. Goran Sedmak and Miloš Judaš acquired funding. All the authors reviewed the manuscript.

## Funding

This research was supported by the Croatian Science Foundation grant no. IP‐2024‐05‐8297 and University of Zagreb grants 10106‐22‐3115 and 10106‐23‐2490. This publication is cofinanced by the Scientific Centre of Excellence for Basic, Clinical and Translational Neuroscience (project “Development of protocol for early diagnosis of hypoxic–ischemic brain lesion in infancy and early childhood”; GA PK1.1.10.0009 funded by the European Union through the European Regional Development Fund).

## Conflicts of Interest

The authors declare no conflicts of interest.

## Data Availability

The data that support the findings of this study are available from the corresponding author upon reasonable request.

## References

[cne70219-bib-0001] Adey, W. R. , D. O. Walter , and D. F. Lindsley . 1962. “Subthalamic Lesions: Effects on Learned Behavior and Correlated Hippocampal and Subcortical Slow‐Wave Activity.” Archives of Neurology 6, no. 3: 194. 10.1001/archneur.1962.00450210022003. 13859405

[cne70219-bib-0002] Akamatsu, W. , H. Fujihara , T. Mitsuhashi , et al. 2005. “The RNA‐Binding Protein HuD Regulates Neuronal Cell Identity and Maturation.” Proceedings of the National Academy of Sciences 102, no. 12: 4625–4630. 10.1073/pnas.0407523102. PMC555491 15764704

[cne70219-bib-0003] Alkemade, A. , G. de Hollander , S. Miletic , et al. 2019. “The Functional Microscopic Neuroanatomy of the Human Subthalamic Nucleus.” Brain Structure and Function 224, no. 9: 3213–3227. 10.1007/s00429-019-01960-3. PMC6875153 31562531

[cne70219-bib-0004] Alkemade, A. , and B. U. Forstmann . 2014. “Do We Need to Revise the Tripartite Subdivision Hypothesis of the human Subthalamic Nucleus (STN)?.” Neuroimage 95: 326–329. 10.1016/j.neuroimage.2014.03.010. 24642281

[cne70219-bib-0005] Araujo, D. J. , A. G. Anderson , S. Berto , et al. 2015. “FoxP1 Orchestration of ASD‐Relevant Signaling Pathways in the Striatum.” Genes & Development 29, no. 20: 2081–2096. 10.1101/gad.267989.115. PMC4617974 26494785

[cne70219-bib-0006] Augood, S. J. , H. J. Waldvogel , M. C. Münkle , R. L. Faull , and P. C. Emson . 1999. “Localization of Calcium‐Binding Proteins and GABA Transporter (GAT‐1) Messenger RNA in the Human Subthalamic Nucleus.” Neuroscience 88, no. 2: 521–534. 10.1016/s0306-4522(98)00226-7. 10197772

[cne70219-bib-0007] Bacon, C. , M. Schneider , C. Le Magueresse , et al. 2015. “Brain‐Specific Foxp1 Deletion Impairs Neuronal Development and Causes Autistic‐Like Behaviour.” Molecular Psychiatry 20, no. 5: 632–639. 10.1038/mp.2014.116. PMC4419151 25266127

[cne70219-bib-0008] Barbier, M. , and P. Y. Risold . 2021. “Understanding the Significance of the Hypothalamic Nature of the Subthalamic Nucleus.” eNeuro 8, no. 5: ENEURO.0116–0121.2021. 10.1523/ENEURO.0116-21.2021. PMC8493884 34518367

[cne70219-bib-0009] Baunez, C. , A. Christakou , Y. Chudasama , C. Forni , and T. W. Robbins . 2007. “Bilateral High‐Frequency Stimulation of the Subthalamic Nucleus on Attentional Performance: Transient Deleterious Effects and Enhanced Motivation in Both Intact and Parkinsonian Rats.” European Journal of Neuroscience 25, no. 4: 1187–1194. 10.1111/j.1460-9568.2007.05373.x. PMC1877866 17331214

[cne70219-bib-0010] Baunez, C. , and S. Lardeux . 2011. “Frontal Cortex‐Like Functions of the Subthalamic Nucleus.” Frontiers in Systems Neuroscience 5: 83. 10.3389/fnsys.2011.00083. PMC3190186 22013416

[cne70219-bib-0011] Baunez, C. , and T. W. Robbins . 1997. “Bilateral Lesions of the Subthalamic Nucleus Induce Multiple Deficits in an Attentional Task in Rats.” European Journal of Neuroscience 9, no. 10: 2086–2099. 10.1111/j.1460-9568.1997.tb01376.x. 9421169

[cne70219-bib-0093] Benarroch, E. E. 2008. “Subthalamic Nucleus and Its Connections.” Neurology 70, no. 21: 1991–1995. 10.1212/01.wnl.0000313022.39329.65. 18490619

[cne70219-bib-0012] Berendse, H. W. , and H. J. Groenewegen . 1991. “The Connections of the Medial Part of the Subthalamic Nucleus in the Rat: Evidence for a Parallel organization.” In Basal Ganglia, III, 89‐98, edited by G. Bernardi , M. B. Carpenter , and G. DiChiara . Plenum Press.

[cne70219-bib-0013] Bertrand, M. , S. Chabardes , V. Fontanier , E. Procyk , J. Bastin , and B. Piallat . 2024. “Contribution of the Subthalamic Nucleus to Motor, Cognitive and Limbic Processes: An Electrophysiological and Stimulation Study in Monkeys.” Frontiers in Neuroscience 18: 1257579. 10.3389/fnins.2024.1257579. PMC10918855 38456146

[cne70219-bib-0092] Bevan, M. D. , J. F. Atherton , and J. Baufreton . 2006. “Cellular Principles Underlying Normal and Pathological Activity in the Subthalamic Nucleus.” Current Opinion in Neurobiology 16, no. 6: 621–628. 10.1016/j.conb.2006.10.003. 17084618

[cne70219-bib-0014] Bokulić, E. , T. Medenica , M. Bobić‐Rasonja , et al. 2025. “The Expression of Transcription Factors in the Human Fetal Subthalamic Nucleus Suggests Its Origin From the First Hypothalamic Prosomere.” Brain Structure and Function 230, no. 2: 33. 10.1007/s00429-025-02893-w. 39831906

[cne70219-bib-0015] Bokulić, E. , T. Medenica , V. Knezović , et al. 2021. “The Stereological Analysis and Spatial Distribution of Neurons in the Human Subthalamic Nucleus.” Frontiers in Neuroanatomy 15: 749390. 10.3389/fnana.2021.749390. PMC8712451 34970124

[cne70219-bib-0016] Bowers, J. M. , M. Perez‐Pouchoulen , N. S. Edwards , and M. M. McCarthy . 2013. “Foxp2 Mediates Sex Differences in Ultrasonic Vocalization by Rat Pups and Directs Order of Maternal Retrieval.” Journal of Neuroscience 33, no. 8: 3276–3283. 10.1523/JNEUROSCI.0425-12.2013. PMC3727442 23426656

[cne70219-bib-0017] Brittain, J. S. , K. E. Watkins , R. A. Joundi , et al. 2012. “A Role for the Subthalamic Nucleus in Response Inhibition During Conflict.” Journal of Neuroscience 32, no. 39: 13396–13401. 10.1523/JNEUROSCI.2259-12.2012. PMC3492743 23015430

[cne70219-bib-0018] Cavalcanti‐Kwiatkoski, R. , R. Raisman‐Vozari , L. Ginestet , and E. Del Bel . 2010. “Altered Expression of Neuronal Nitric Oxide Synthase in Weaver Mutant Mice.” Brain Research 1326: 40–50. 10.1016/j.brainres.2010.02.056. 20219442

[cne70219-bib-0019] Cha, C. I. , S. G. Sohn , Y. H. Chung , C. Shin , and S. H. Baik . 2000. “Region‐Specific Changes of NOS‐IR Cells in the Basal Ganglia of the Aged Rat.” Brain Research 854, no. 1–2: 239–244. 10.1016/s0006-8993(99)02279-9. 10784129

[cne70219-bib-0020] Chachlaki, K. , and V. Prevot . 2019. “Nitric Oxide Signalling in the Brain and Its Control of Bodily Functions.” British Journal of Pharmacology 177, no. 24: 5437–5458. 10.1111/bph.14800. PMC7707094 31347144

[cne70219-bib-0021] Chasse, R. , R. McLeod , A. Surian , R. H. Fitch , and J. Li . 2024. “The Role of Cerebellar FOXP1 in the Development of Motor and Communicative Behaviors in Mice.” Genes, Brain and Behavior 23, no. 5: e70001. 10.1111/gbb.70001. PMC11479947 39407418

[cne70219-bib-0022] Choi, B. S. , G. Shen , G. Nan , J. M. Kim , K. Y. Jung , and B. Jeon . 2018. “Dramatic Psychiatric and Behavioral Symptoms Following a Subthalamic Lesion.” Journal of Clinical Neuroscience 47: 154–156. 10.1016/j.jocn.2017.10.051. 29113860

[cne70219-bib-0023] Chudasama, Y. , C. Baunez , and T. W. Robbins . 2003. “Functional Disconnection of the Medial Prefrontal Cortex and Subthalamic Nucleus in Attentional Performance: Evidence for Corticosubthalamic Interaction.” Journal of Neuroscience 23, no. 13: 5477–5485. 10.1523/JNEUROSCI.23-13-05477.2003. PMC6741240 12843247

[cne70219-bib-0024] Co, M. , A. G. Anderson , and G. Konopka . 2020. “FOXP Transcription Factors in Vertebrate Brain Development, Function, and Disorders.” WIREs Developmental Biology 9, no. 5: e375. 10.1002/wdev.375. PMC8286808 31999079

[cne70219-bib-0025] Czarnecka, A. , T. Lenda , H. Domin , J. Konieczny , M. Śmiałowska , and E. Lorenc‐Koci . 2013. “Alterations in the Expression of nNOS in the Substantia Nigra and Subthalamic Nucleus of 6‐OHDA‐Lesioned Rats: the Effects of Chronic Treatment With L‐DOPA and the Nitric Oxide Donor, Molsidomine.” Brain Research 1541: 92–105. 10.1016/j.brainres.2013.10.011. 24129225

[cne70219-bib-0026] Dasen, J. S. , A. De Camilli , B. Wang , P. W. Tucker , and T. M. Jessell . 2008. “Hox Repertoires for Motor Neuron Diversity and Connectivity Gated by a Single Accessory Factor, FoxP1.” Cell 134, no. 2: 304–316. 10.1016/j.cell.2008.06.019. 18662545

[cne70219-bib-0027] De Vente, J. , D. A. Hopkins , M. Markerink‐Van Ittersum , P. C. Emson , H. H. Schmidt , and H. W. Steinbusch . 1998. “Distribution of Nitric Oxide Synthase and Nitric Oxide‐Receptive, Cyclic GMP‐Producing Structures in the Rat Brain.” Neuroscience 87, no. 1: 207–241. 10.1016/s0306-4522(98)00171-7. 9722153

[cne70219-bib-0088] DeLong, M. R. , M. D. Crutcher , and A. P. Georgopoulos . 1985. “Primate Globus Pallidus and Subthalamic Nucleus: Functional Organization.” Journal of Neurophysiology 53, no. 2: 530–543. 10.1152/jn.1985.53.2.530. 3981228

[cne70219-bib-0028] Dudás, B. 2021. “Anatomy and Cytoarchitectonics of the Human Hypothalamus.” Handbook of Clinical Neurology 179: 45–66. 10.1016/B978-0-12-819975-6.00001-7. 34225981

[cne70219-bib-0029] Ellenbroek, B. , and J. Youn . 2016. “Rodent Models in Neuroscience Research: Is It a Rat Race?.” Disease Models & Mechanisms 9, no. 10: 1079–1087. 10.1242/dmm.026120. PMC5087838 27736744

[cne70219-bib-0030] Endoh, M. , K. Maiese , and J. A. Wagner . 1994. “Expression of the Neural Form of Nitric Oxide Synthase by CA1 Hippocampal Neurons and Other Central Nervous System Neurons.” Neuroscience 63, no. 3: 679–689. 10.1016/0306-4522(94)90514-2. 7534883

[cne70219-bib-0031] Eve, D. J. , A. P. Nisbet , A. E. Kingsbury , et al. 1998. “Basal Ganglia Neuronal Nitric Oxide Synthase mRNA Expression in Parkinson's Disease.” Molecular Brain Research 63, no. 1: 62–71. 10.1016/s0169-328x(98)00259-9. 9838046

[cne70219-bib-0032] Ferland, R. J. , T. J. Cherry , P. O. Preware , E. E. Morrisey , and C. A. Walsh . 2003. “Characterization of Foxp2 and Foxp1 mRNA and Protein in the Developing and Mature Brain.” Journal of Comparative Neurology 460, no. 2: 266–279. 10.1002/cne.10654. 12687690

[cne70219-bib-0033] Frank, M. J. , J. Samanta , A. A. Moustafa , and S. J. Sherman . 2007. “Hold Your Horses: Impulsivity, Deep Brain Stimulation, and Medication in Parkinsonism.” Science 318, no. 5854: 1309–1312. 10.1126/science.1146157. 17962524

[cne70219-bib-0034] Gage, G. J. , D. R. Kipke , and W. Shain . 2012. “Whole Animal Perfusion Fixation for Rodents.” Journal of Visualized Experiments: JoVE 65, no. 65: 3564. 10.3791/3564. PMC3476408 22871843

[cne70219-bib-0094] Greenhouse, I. , S. Gould , M. Houser , G. Hicks , J. Gross , and A. R. Aron . 2011. “Stimulation at Dorsal and Ventral Electrode Contacts Targeted at the Subthalamic Nucleus Has Different Effects on Motor and Emotion Functions in Parkinson's Disease.” Neuropsychologia 49, no. 3: 528–534. 10.1016/j.neuropsychologia.2010.12.030. 21184765

[cne70219-bib-0035] Grillner, S. , and B. Robertson . 2016. “The Basal Ganglia Over 500 Million Years.” Current Biology 26, no. 20: R1088–R1100. 10.1016/j.cub.2016.06.041. 27780050

[cne70219-bib-0036] Groenewegen, H. J. , and H. W. Berendse . 1990. “Connections of the Subthalamic Nucleus With Ventral Striatopallidal Parts of the Basal Ganglia in the Rat.” Journal of Comparative Neurology 294, no. 4: 607–622. 10.1002/cne.902940408. 2341628

[cne70219-bib-0090] Hamani, C. 2004. “The Subthalamic Nucleus in the Context of Movement Disorders.” Brain 127, no. 1: 4–20. 10.1093/brain/awh029. 14607789

[cne70219-bib-0037] Hardman, C. D. , J. M. Henderson , D. I. Finkelstein , M. K. Horne , G. Paxinos , and G. M. Halliday . 2002. “Comparison of the Basal Ganglia in Rats, Marmosets, Macaques, Baboons, and Humans: Volume and Neuronal Number for the Output, Internal Relay, and Striatal Modulating Nuclei.” Journal of Comparative Neurology 445, no. 3: 238–255. 10.1002/cne.10165. 11920704

[cne70219-bib-0089] Joel, D. , and I. Weiner . 1997. “The Connections of the Primate Subthalamic Nucleus: Indirect Pathways and the Open‐interconnected Scheme of Basal Ganglia‐thalamocortical Circuitry.” Brain Research Reviews 23, no. 1‐2: 62–78. 10.1016/s0165-0173(96)00018-5. 9063587

[cne70219-bib-0038] Judaš, M. , G. Šimić , Z. Petanjek , et al. 2011. “The Zagreb Collection of Human Brains: A Unique, Versatile, but Underexploited Resource for the Neuroscience Community.” Annals of the New York Academy of Sciences 1225, no. suppl 1: E105–E130. 10.1111/j.1749-6632.2011.05993.x. 21599691

[cne70219-bib-0095] Keuken, M. C. , H. B. M. Uylings , S. Geyer , A. Schäfer , R. Turner , and B. U. Forstmann . 2012. “Are There Three Subdivisions in the Primate Subthalamic Nucleus?” Frontiers in Neuroanatomy 6: 14. 10.3389/fnana.2012.00014. PMC3349268 22590455

[cne70219-bib-0039] Kita, H. , and S. T. Kitai . 1987. “Efferent Projections of the Subthalamic Nucleus in the Rat: Light and Electron Microscopic Analysis With the PHA‐L Method.” Journal of Comparative Neurology 260, no. 3: 435–452. 10.1002/cne.902600309. 2439552

[cne70219-bib-0040] Kondabolu, K. , N. M. Doig , O. Ayeko , et al. 2023. “A Selective Projection From the Subthalamic Nucleus to Parvalbumin‐Expressing Interneurons of the Striatum.” eNeuro 10, no. 7: ENEURO.0417–0421.2023. 10.1523/ENEURO.0417-21.2023. PMC10327533 37364998

[cne70219-bib-0043] Lévesque, J. C. , and A. Parent . 2005. “GABAergic Interneurons in Human Subthalamic Nucleus.” Movement Disorders 20, no. 5: 574–584. 10.1002/mds.20374. 15645534

[cne70219-bib-0041] Lange, H. , G. Thörner , A. Hopf , and K. F. Schröder . 1976. “Morphometric Studies of the Neuropathological Changes in Choreatic Diseases.” Journal of the Neurological Sciences 28, no. 4: 401–425. 10.1016/0022-510x(76)90114-3. 133209

[cne70219-bib-0042] Lardeux, S. , D. Paleressompoulle , R. Pernaud , M. Cador , and C. Baunez . 2013. “Different Populations of Subthalamic Neurons Encode Cocaine Vs. Sucrose Reward and Predict Future Error.” Journal of Neurophysiology 110, no. 7: 1497–1510. 10.1152/jn.00160.2013. 23864369

[cne70219-bib-0097] Lardeux, S. , R. Pernaud , D. Paleressompoulle , and C. Baunez . 2009. “Beyond the Reward Pathway: Coding Reward Magnitude and Error in the Rat Subthalamic Nucleus.” Journal of Neurophysiology 102, no. 4: 2526–2537. 10.1152/jn.91009.2008. 19710371

[cne70219-bib-0044] Lindsley, D. F. , R. J. Barton , and R. J. Atkins . 1970. “Effects of Subthalamic Lesions on Peripheral and Central Arousal Thresholds in Cats.” Experimental Neurology 26, no. 1: 109–119. 10.1016/0014-4886(70)90092-0. 5411017

[cne70219-bib-0045] Lozano, R. , C. Gbekie , P. M. Siper , et al. 2021. “FOXP1 Syndrome: A Review of the Literature and Practice Parameters for Medical Assessment and Monitoring.” Journal of Neurodevelopmental Disorders 13, no. 1: 18. 10.1186/s11689-021-09358-1. PMC8066957 33892622

[cne70219-bib-0046] Luijerink, L. , K. A. Waters , and R. Machaalani . 2021. “Immunostaining for NeuN Does Not Show All Mature and Healthy Neurons in the Human and Pig Brain: Focus on the Hippocampus.” Applied Immunohistochemistry & Molecular Morphology 29, no. 6: e46–e56. 10.1097/PAI.0000000000000925. 33710124

[cne70219-bib-0047] Mai, J. K. , J. Assheuer , and G. Paxinos . 2004. Atlas of the Human Brain. 2nd ed. Academic Press, Elsevier.

[cne70219-bib-0085] Mallet, L. , M. Polosan , N. Jaafari , et al. 2008. “Subthalamic Nucleus Stimulation in Severe Obsessive–Compulsive Disorder.” New England Journal of Medicine 359, no. 20: 2121–2134. 10.1056/nejmoa0708514. 19005196

[cne70219-bib-0048] Mannella, P. , A. M. Sanchez , M. S. Giretti , A. R. Genazzani , and T. Simoncini . 2009. “Oestrogen and Progestins Differently Prevent Glutamate Toxicity in Cortical Neurons Depending on Prior Hormonal Exposure via the Induction of Neural Nitric Oxide Synthase.” Steroids 74, no. 8: 650–656. 10.1016/j.steroids.2009.02.011. 19463685

[cne70219-bib-0049] Marusich, M. F. , H. M. Furneaux , P. D. Henion , and J. A. Weston . 1994. “Hu Neuronal Proteins Are Expressed in Proliferating Neurogenic Cells.” Journal of Neurobiology 25, no. 2: 143–155. 10.1002/neu.480250206. 7517436

[cne70219-bib-0050] Meerschaut, I. , D. Rochefort , N. Revençu , et al. 2017. “FOXP1‐Related Intellectual Disability Syndrome: A Recognisable Entity.” Journal of Medical Genetics 54, no. 9: 613–623. 10.1136/jmedgenet-2017-104579. 28735298

[cne70219-bib-0051] Merchan‐Sala, P. , D. Nardini , R. R. Waclaw , and K. Campbell . 2017. “Selective Neuronal Expression of the SoxE Factor, Sox8, in Direct Pathway Striatal Projection Neurons of the Developing Mouse Brain.” Journal of Comparative Neurology 525, no. 13: 2805–2819. 10.1002/cne.24232. PMC6432783 28472858

[cne70219-bib-0052] Miletić, S. , M. C. Keuken , M. J. Mulder , R. Trampel , G. de Hollander , and B. U. Forstmann . 2022. “7T Functional MRI Finds no Evidence for Distinct Functional Subregions in the Subthalamic Nucleus During a Speeded Decision‐Making Task.” Cortex; A Journal Devoted to the Study of the Nervous System and Behavior 155: 162–188. 10.1016/j.cortex.2022.06.014. 35994782

[cne70219-bib-0053] Nisbet, A. P. , O. J. Foster , A. Kingsbury , A. J. Lees , and C. D. Marsden . 1994. “Nitric Oxide Synthase mRNA Expression in Human Subthalamic Nucleus, Striatum and Globus Pallidus: Implications for Basal Ganglia Function.” Molecular Brain Research 22, no. 1–4: 329–332. 10.1016/0169-328x(94)90062-0. 7516995

[cne70219-bib-0054] Oorschot, D. E. 1996. “Total Number of Neurons in the Neostriatal, Pallidal, Subthalamic, and Substantia Nigral Nuclei of the Rat Basal Ganglia: A Stereological Study Using the Cavalieri and Optical Disector Methods.” Journal of Comparative Neurology 366, no. 4: 580–599. 10.1002/(SICI)1096-9861(19960318)366:4<;580::AID-CNE3>;3.0.CO;2-0. 8833111

[cne70219-bib-0055] Palmesino, E. , D. L. Rousso , T. J. Kao , et al. 2010. “Foxp1 and lhx1 Coordinate Motor Neuron Migration With Axon Trajectory Choice by Gating Reelin Signalling.” PLoS Biology 8, no. 8: e1000446. 10.1371/journal.pbio.1000446. PMC2919418 20711475

[cne70219-bib-0083] Papuć, E. , T. Trojanowski , K. Obszanska , and Z. Stelmasiak . 2014. “Aggressive Behavior as a Rare Side Effect of Subthalamic Stimulation in Parkinson's Disease.” Neurocase 21, no. 2: 220–225. 10.1080/13554794.2014.890729. 24564255

[cne70219-bib-0056] Parent, A. , and L. N. Hazrati . 1995. “Functional Anatomy of the Basal Ganglia. II. The Place of Subthalamic Nucleus and External Pallidium in Basal Ganglia Circuitry.” Brain Research Reviews 20, no. 1: 128–154. 10.1016/0165-0173(94)00008-d. 7711765

[cne70219-bib-0084] Parolari, L. , M. Schneeberger , N. Heintz , and J. M. Friedman . 2021. “Functional Analysis of Distinct Populations of Subthalamic Nucleus Neurons on Parkinson's Disease and OCD‐Like Behaviors in Mice.” Molecular Psychiatry 26, no. 11: 7029–7046. 10.1038/s41380-021-01162-6. 34099874

[cne70219-bib-0057] Pascale, A. , M. Amadio , and A. Quattrone . 2008. “Defining a Neuron: Neuronal ELAV Proteins.” Cellular and Molecular Life Sciences 65, no. 1: 128–140. 10.1007/s00018-007-7017-y. PMC11131659 17928954

[cne70219-bib-0058] Paxinos, G. , and K. Franklin . 2004. The Mouse Brain in Stereotaxic Coordinates. 2nd ed. Academic Press, Elsevier.

[cne70219-bib-0059] Paxinos, G. , and C. Watson . 2007. The Rat Brain in Stereotaxic Coordinates. 6th ed. Academic Press, Elsevier.

[cne70219-bib-0060] Polosan, M. , F. Droux , A. Kibleur , et al. 2019. “Affective Modulation of the Associative‐Limbic Subthalamic Nucleus: Deep Brain Stimulation in Obsessive–Compulsive Disorder.” Translational Psychiatry 9: 73. 10.1038/s41398-019-0404-y. PMC6361948 30718450

[cne70219-bib-0096] Prasad, A. A. , and Å. Wallén‐Mackenzie . 2024. “Architecture of the Subthalamic Nucleus.” Communications Biology 7, no. 1: 78. 10.1038/s42003-023-05691-4. PMC10782020 38200143

[cne70219-bib-0086] Rodrigo, J. , D. R. Springall , O. Uttenthal , et al. 1994. “Localization of Nitric Oxide Synthase in the Adult Rat Brain.” Philosophical Transactions of the Royal Society of London. Series B: Biological Sciences 345, no. 1312: 175–221. 10.1098/rstb.1994.0096. 7526408

[cne70219-bib-0091] Romanelli, P. , V. Esposito , D. W. Schaal , and G. Heit . 2005. “Somatotopy in the Basal Ganglia: Experimental and Clinical Evidence for Segregated Sensorimotor Channels.” Brain Research Reviews 48, no. 1: 112–128. 10.1016/j.brainresrev.2004.09.00. 15708631

[cne70219-bib-0061] Ruze, A. , B. Wang , J. Jin , P. Hou , D. Tuerxun , and S. Amuti . 2023. “Bradykinin B1 Receptor Antagonist Protects Against Cold Stress–Induced Erectile Dysfunction in Rats.” Sexual Medicine 11, no. 1: qfac004. 10.1093/sexmed/qfac004. PMC10065187 37007851

[cne70219-bib-0062] Santos‐Lobato, B. L. , E. Del‐Bel , J. E. Pittella , and V. Tumas . 2016. “Cytoarchitecture of Nitrergic Neurons in the Human Striatum and Subthalamic Nucleus.” Brain Research Bulletin 124: 129–135. 10.1016/j.brainresbull.2016.04.003. 27060610

[cne70219-bib-0063] Saper, C. B. 2012. “Hypothalamus.” In The Human Nervous System, 3rd ed. edited by J. K. Mai and G. Paxinos , 548–583. Academic Press. 10.1016/B978-0-12-374236-0.10016-1.

[cne70219-bib-0087] Sardo, P. , G. Ferraro , F. Carletti , S. D'Agostino , and V. La Grutta . 2006. “The Discharge of Subthalamic Neurons Is Modulated by Inhibiting the Nitric Oxide Synthase in the Rat.” Neuroscience Letters 396, no. 3: 252–256. 10.1016/j.neulet.2005.11.054. 16380210

[cne70219-bib-0064] Sensi, M. , R. Eleopra , M. A. Cavallo , et al. 2004. “Explosive‐Aggressive Behavior Related to Bilateral Subthalamic Stimulation.” Parkinsonism & Related Disorders 10, no. 4: 247–251. 10.1016/j.parkreldis.2004.01.007. 15120100

[cne70219-bib-0065] Sheng, H. , X. Li , and Y. Xu . 2019. “Knockdown of FOXP1 Promotes the Development of Lung Adenocarcinoma.” Cancer Biology & Therapy 20, no. 4: 537–545. 10.1080/15384047.2018.1537999. PMC6422458 30409062

[cne70219-bib-0066] Shi, H. J. , D. L. Wu , R. Chen , N. Li , and L. J. Zhu . 2022. “Requirement of Hippocampal DG nNOS‐CAPON Dissociation for the Anxiolytic and Antidepressant Effects of Fluoxetine.” Theranostics 12, no. 8: 3656–3675. 10.7150/thno.70370. PMC9131266 35664081

[cne70219-bib-0067] Siper, P. M. , S. De Rubeis , M. D. P. Trelles , et al. 2017. “Prospective Investigation of FOXP1 Syndrome.” Molecular Autism 8: 57. 10.1186/s13229-017-0172-6. PMC5655854 29090079

[cne70219-bib-0068] Skidmore, J. M. , J. D. Cramer , J. F. Martin , and D. M. Martin . 2008. “Cre Fate Mapping Reveals Lineage Specific Defects in Neuronal Migration With Loss of Pitx2 Function in the Developing Mouse Hypothalamus and Subthalamic Nucleus.” Molecular and Cellular Neuroscience 37, no. 4: 696–707. 10.1016/j.mcn.2007.12.015. PMC2386145 18206388

[cne70219-bib-0069] Tamura, S. , Y. Morikawa , H. Iwanishi , T. Hisaoka , and E. Senba . 2004. “Foxp1 Gene Expression in Projection Neurons of the Mouse Striatum.” Neuroscience 124, no. 2: 261–267. 10.1016/j.neuroscience.2003.11.036. 14980377

[cne70219-bib-0070] Temel, Y. , A. Blokland , H. W. Steinbusch , and V. Visser‐Vandewalle . 2005. “The Functional Role of the Subthalamic Nucleus in Cognitive and Limbic Circuits.” Progress in Neurobiology 76, no. 6: 393–413. 10.1016/j.pneurobio.2005.09.005. 16249050

[cne70219-bib-0071] Teramitsu, I. , L. C. Kudo , S. E. London , D. H. Geschwind , and S. A. White . 2004. “Parallel FoxP1 and FoxP2 Expression in Songbird and Human Brain Predicts Functional Interaction.” Journal of Neuroscience 24, no. 13: 3152–3163. 10.1523/JNEUROSCI.5589-03.2004. PMC6730014 15056695

[cne70219-bib-0072] Tinterri, A. , M. Deck , M. Keita , et al. 2018. “Tangential Migration of Corridor Guidepost Neurons Contributes to Anxiety Circuits.” Journal of Comparative Neurology 526, no. 3: 397–411. 10.1002/cne.24330. 28921616

[cne70219-bib-0073] Tricoire, L. , and T. Vitalis . 2012. “Neuronal Nitric Oxide Synthase Expressing Neurons: A Journey From Birth to Neuronal Circuits.” Frontiers in Neural Circuits 6: 82. 10.3389/fncir.2012.00082. PMC3514612 23227003

[cne70219-bib-0074] Tulloch, A. J. , S. Teo , B. V. Carvajal , M. Tessier‐Lavigne , and A. Jaworski . 2019. “Diverse Spinal Commissural Neuron Populations Revealed by Fate Mapping and Molecular Profiling Using a Novel Robo3Cre Mouse.” Journal of Comparative Neurology 527, no. 18: 2948–2972. 10.1002/cne.24720. PMC6790177 31152445

[cne70219-bib-0075] Vincent, S. R. , and H. Kimura . 1992. “Histochemical Mapping of Nitric Oxide Synthase in the Rat Brain.” Neuroscience 46, no. 4: 755–784. 10.1016/0306-4522(92)90184-4. 1371855

[cne70219-bib-0076] Voon, V. , F. Droux , L. Morris , et al. 2017. “Decisional Impulsivity and the Associative‐Limbic Subthalamic Nucleus in Obsessive‐Compulsive Disorder: Stimulation and Connectivity.” Brain 140, no. 2: 442–456. 10.1093/brain/aww309. PMC5278307 28040671

[cne70219-bib-0077] Wallén‐Mackenzie, Å. , S. Dumas , M. Papathanou , et al. 2020. “Spatio‐Molecular Domains Identified in the Mouse Subthalamic Nucleus and Neighboring Glutamatergic and GABAergic Brain Structures.” Communications Biology 3, no. 1: 338. 10.1038/s42003-020-1028-8. PMC7334224 32620779

[cne70219-bib-0078] Wang, B. , T. Lufkin , and J. L. Rubenstein . 2011. “Dlx6 Regulates Molecular Properties of the Striatum and Central Nucleus of the Amygdala.” Journal of Comparative Neurology 519, no. 12: 2320–2334. 10.1002/cne.22618. PMC4019376 21452241

[cne70219-bib-0079] Wang, B. , J. Weidenfeld , M. M. Lu , et al. 2004. “Foxp1 Regulates Cardiac Outflow Tract, Endocardial Cushion Morphogenesis and Myocyte Proliferation and Maturation.” Development 131, no. 18: 4477–4487. 10.1242/dev.01287. 15342473

[cne70219-bib-0080] West, A. R. , M. P. Galloway , and A. A. Grace . 2002. “Regulation of Striatal Dopamine Neurotransmission by Nitric Oxide: Effector Pathways and Signaling Mechanisms.” Synapse 44, no. 4: 227–245. 10.1002/syn.10076. 11984858

[cne70219-bib-0081] Xia, Y. , and T. L. Krukoff . 2004. “Estrogen Induces Nitric Oxide Production via Activation of Constitutive Nitric Oxide Synthases in Human Neuroblastoma Cells.” Endocrinology 145, no. 10: 4550–4557. 10.1210/en.2004-0327. 15242984

[cne70219-bib-0082] Zwirner, J. , D. Möbius , I. Bechmann , et al. 2017. “Subthalamic Nucleus Volumes Are Highly Consistent but Decrease Age‐Dependently – A Combined Magnetic Resonance Imaging and Stereology Approach in Humans.” Human Brain Mapping 38, no. 2: 909–922. 10.1002/hbm.23427. PMC6867036 27726278

